# The Customer Isn't Always Right—Conservation and Animal Welfare Implications of the Increasing Demand for Wildlife Tourism

**DOI:** 10.1371/journal.pone.0138939

**Published:** 2015-10-21

**Authors:** Tom P. Moorhouse, Cecilia A. L. Dahlsjö, Sandra E. Baker, Neil C. D'Cruze, David W. Macdonald

**Affiliations:** 1 Wildlife Conservation Research Unit, Department of Zoology, University of Oxford, Recanati-Kaplan Centre, Tubney, United Kingdom; 2 World Animal Protection (formerly the World Society for the Protection of Animals), London, United Kingdom; University of New South Wales, AUSTRALIA

## Abstract

Tourism accounts for 9% of global GDP and comprises 1.1 billion tourist arrivals per annum. Visits to wildlife tourist attractions (WTAs) may account for 20–40% of global tourism, but no studies have audited the diversity of WTAs and their impacts on the conservation status and welfare of subject animals. We scored these impacts for 24 types of WTA, visited by 3.6–6 million tourists per year, and compared our scores to tourists’ feedback on TripAdvisor. Six WTA types (impacting 1,500–13,000 individual animals) had net positive conservation/welfare impacts, but 14 (120,000–340,000 individuals) had negative conservation impacts and 18 (230,000–550,000 individuals) had negative welfare impacts. Despite these figures only 7.8% of all tourist feedback on these WTAs was negative due to conservation/welfare concerns. We demonstrate that WTAs have substantial negative effects that are unrecognised by the majority of tourists, suggesting an urgent need for tourist education and regulation of WTAs worldwide.

## Introduction

Tourism is a major global economic driver which in 2013 was worth over a trillion US dollars, accounted for 9% of global GDP, and provided 1 in 11 jobs worldwide [1]. International tourist arrivals have continually increased from 25 million in 1950 to 1087 million in 2013, with 1.8 billion predicted by 2030 [1]. Although there are no reliable global measures of the economic impact of wildlife tourism (tourism specifically based on encounters with non-domesticated animals) [2], it is the leading foreign exchange earner in several countries [3] and attending wildlife tourist attractions (WTAs) is a prime tourist motivation [2]. For example in 2006 approximately 2.2 million of Australia’s inbound tourists visited WTAs, representing 43% of all their international tourists [4], and one study concluded that wildlife tourism in 1988 accounted for 20–40 percent of international tourism globally [5]. Wildlife tourism represents a significant proportion of a huge global market that is predicted to increase in the coming decades.

WTAs are extremely diverse, but can be divided into four broad categories [2]: wildlife-watching tourism (viewing or otherwise interacting with free-ranging animals); captive-wildlife tourism (viewing animals in human-made confinement; principally zoos, wildlife parks, animal sanctuaries and aquaria, but also includes circuses and shows by mobile wildlife exhibitors); hunting tourism; fishing tourism. These types of wildlife tourism are either non-consumptive, e.g. bird watching, whale and dolphin watching, aquariums and wildlife parks [6], or consumptive—involving animals being deliberately killed or removed, or having their body parts used [7]—e.g. hunting and fishing [2].

WTAs can provide opportunities and livelihoods for the local human population [8] and can also secure long-term conservation of wildlife and wildlife habitats [2, 4, 6] through practical conservation efforts by volunteers and operators, the creation of local socio-economic incentives for the preservation of wildlife and their habitats [9, 10], and tourist education, which may promote positive attitudes towards species preservation and animal welfare, and increase future conservation revenue through future philanthropic donations [11–13]. Conversely, improperly managed WTAs can have an array of negative impacts on both the conservation and welfare status of subject taxa and individuals, whether in the wild or captivity [2, 3]. These impacts include removal of individuals from wild populations, injury, disease and death [2, 14], short- and long-term animal behavioural changes [14–18], stress and aberrant physiological responses [2, 3, 19–21], altered feeding and reproductive behaviour [3, 16, 22] and habitat alteration / loss [3, 14].

All WTAs at least partially trade-off values of conservation, animal welfare, visitor satisfaction and profitability [3, 20]. Tourists’ individual motivations and awareness will determine what they are willing to accept [3, 23, 24], but WTAs may have impacts that are difficult to detect [25] and some may foster a deliberate disconnect between their stated conservation or welfare credentials, and what they deliver in practice [26, 27]. Given the recent—and expected future—global increases in wildlife tourism, there is a pressing need to audit the diversity of WTAs and their impacts, positive, neutral or negative, on the conservation and welfare status of the animals involved, and to understand tourists' perceptions of WTAs in relation to an objective assessment of their impacts [3], to highlight areas in which tourist education may be beneficial.

A number of recent studies has reviewed the impacts of individual WTA types [15, 25, 26], but no attempt has been made to describe the diversity of WTAs and their impacts worldwide. Similarly, while studies have examined levels of visitor satisfaction and educational engagement for individual WTAs [4, 6, 12, 13, 24, 28] tourist feedback has not been related across a number of WTA types to any independent assessment of those WTAs’ conservation and welfare consequences.

We here present a preliminary audit of the types of non-consumptive, non-zoo WTAs that currently exist worldwide, and a conceptual framework within which to categorise them. Within each of our identified categories we select a subset of WTA types for objective review of their welfare and conservation impacts. We then examine the extent to which tourists are adequate assessors of these impacts by analysing feedback reviews of wildlife tourism attractions, left by customers on TripAdvisor—the largest global internet review website, hosting sites in 45 countries and 28 languages, with > 480,000 tourist attractions rated [29]—comparing these with our independent review for each type of attraction.

## Materials and Methods

We excluded purely consumptive WTAs (hunting and fishing) because the tourists attending are likely to have anticipated, and accepted, direct impacts on the subject wildlife. We also excluded zoos, first because their long history [30] means that tourists are likely to be familiar with their impacts, second because limited project time required prioritisation of the relatively unstudied and unfamiliar non-zoo WTAs, and third because zoos typically host a diversity of captive wildlife, whereas non-zoo WTAs usually comprise only one or two subject taxa, with which tourists specifically desire an encounter. Similarly we also exclude wildlife in national parks and protected areas as these typically host multiple taxa and visitors do not anticipate direct encounters with the animals.

### Rationale, approach and limitations

The desired outputs from this study were an audit of existing types of WTAs, an assessment of their conservation and welfare impacts, and an analysis of tourists’ attitudes towards them. A given WTA type could comprise a single attraction (albeit potentially, but not necessarily, with large annual numbers of visitors and /or subject animals) or several hundred individual attractions. In practice, therefore, conservation and welfare impact assessments were typically made across a number of individual institutions, acknowledging that standards may vary between them. The quality and quantity of information pertaining to these impacts (e.g. the number of animals involved, the conditions in which they were maintained) varied between WTA types, and sufficient, credible sources were not available in all cases. We therefore adopted a protocol to ensure a representative selection across the full range of identified WTA types.

First we produced as extensive a list of WTA types as possible, using information from peer reviewed articles, grey literature (including reports by conservation and welfare NGOs and non-profit organisations, as well as newspaper articles), and online sources (including advocacy websites, and WTA promotional websites). We grouped these WTA types into categories (e.g. Wild Attractions; S1 Table), and selected five (where possible) representative example WTA types from each category (e.g. captive dolphin interactions, see S2 Table; Table 1), for which sufficient data were available. The conservation and welfare impacts of these example WTA types were scored using information from the data sources listed above. Where possible the original research and reports were traced and cited, and in cases where estimates differed between sources we prioritised evidence from the academic literature and reports by NGO and non-profit bodies over information hosted on advocacy or WTA promotional websites. Tourists’ assessments for each of the selected examples were collated from online reviews from the largest travel review website, TripAdvisor, for every specific institution within a given WTA type (details below).

**Table 1:** Conservation and welfare scores, accessibility (number of visitors per annum and number of animals held) and tourist dissatisfaction score (percentage of reviews on TripAdvisor that were negative for WTAs within a given type) for 24 representative WTA types, selected across five categories of WTA. See Fig 1A and 1B, and S3A Table–S3X Table and S4A Table–S4X Table for score derivation and supporting references, respectively.

WTA type	Conservation score	Welfare score	Tourist dissatisfaction score (% negative reviews)	Number of visitors annually	Number of animals in the attraction
**Captive interactions**					
Bear parks (Japan only)	-1	-3	2.8 (4.0, n = 2)	100,000–500,000	100–1000
Dolphin interactions (captive)	-1	-2	3.5 (7.6, n = 46)	>500,000	1000–10,000
Elephant parks / treks	-2	-2	12.8 (17.6, n = 55)	>500,000	10,000–100,000
Tiger interactions	-1	-3	16.4 (11.9, n = 3)	100,000–500,000	100–1,000
Lion encounters	+1	-1	7.0 (6.8, n = 9)	100,000–500,000	1,000–10,000
**Sanctuary attractions**					
Bear sanctuary	+1	+2	0.0 (0.0, n = 2)	>500,000	1000–10,000
Elephant sanctuary	+1	+2	0.3 (0.5, n = 7)	1,000–10,000	100–1,000
Lion sanctuary	+1	+2	0.0 (n = 1)	10,000–100,000	100–1,000
Orang-utan sanctuary	+3	+2	6.0 (11.9, n = 4)	10,000–100,000	100–1,000
Dolphin sanctuary	+1	+2	-	100,000–500,000	<100
**Farmed wildlife attractions**					
Civet coffee	-1	-3	20.0 (n = 1)	-	1,000–10,000
Sea turtle farm	+1	-3	20.0 (18.0, n = 4)	>500,000	10,000–100,000
Tiger farms	-1	-3	-	1000–10,000	1000–10,000
Crocodile farms	+1	-2	24.1 (26.6, n = 14)	>500,000	>100,000
Bear bile farming	-2	-3	-	>500,000	10,000–100,000
**Street performance**					
Street dancing macaques	-1	-2	-	1000–10,000	100–1000
Hyena men in Nigeria	-1	-2	-	1000–10,000	<100
Snake charming	-2	-3	-	100,000–500,000	100–1000
Bear dancing	-2	-3	-	1000–10,000	100–1000
**Wild attractions**					
Dolphin interactions (wild)	-2	-2	0.9 (3.3, n = 23)	10,000–100,000	>100,000
Gorilla trekking	+3	-1	0.0 (0.0, n = 2)	10,000–100,000	100–1000
Gibbon watching	+3	0	-	1000–10,000	<100
Shark cage diving	-2	-2	0.2 (0.5, n = 15)	>500,000	100–1000
Polar bear sightseeing	-1	-2	-	1000–10,000	100–1000

### Attribution of conservation and welfare impact scores

**Fig 1:**
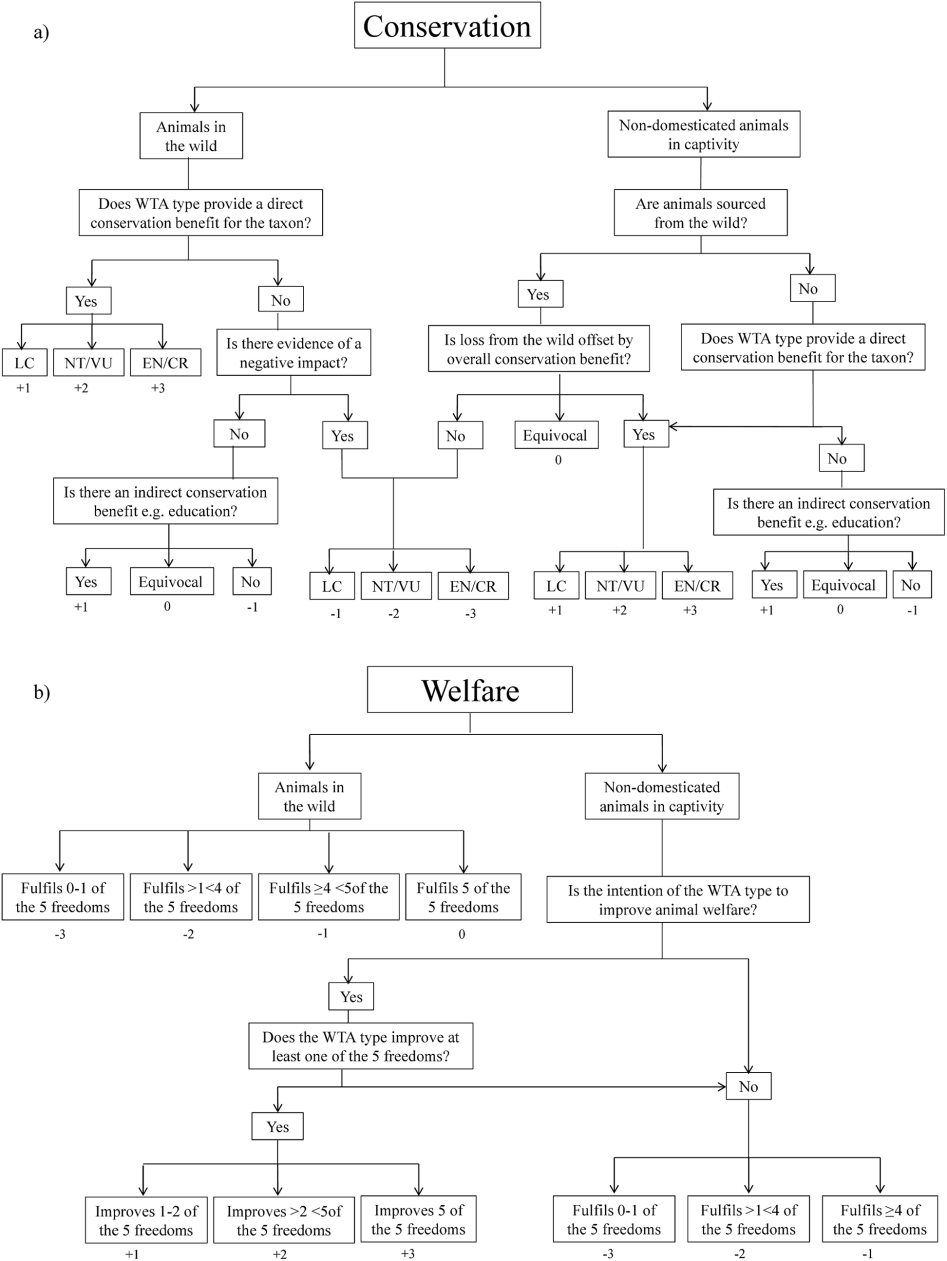
Flow charts detailing the logic underpinning the allocation of a) conservation scores and b) welfare scores to types of wildlife tourist attractions (WTA types). Final scores range from -3 to +3 and are indicated below the relevant boxes. LC, NT, VU, EN, CR indicate the IUCN Redlist status of the species (Least Concern, Near Threatened, Vulnerable, Endangered, Critically Endangered). Please see accompanying information in S1 Appendix.

The conservation and welfare impacts for each WTA type were ranked separately using a seven point scale (+1 to +3 for positive impacts, -1 to -3 for negative impacts, and 0 for no impact). This scale was a compromise between obtaining useful separation between attractions on each axis (welfare and conservation), but not implying precision beyond that available in the source material. Scores were awarded in response to the logic outlined in Fig 1A and 1B (detailed, accompanying rationale is provided in S1 Appendix), with data, analysis and supporting references for each WTA type entered into standardised pro-forma tables (see S3A Table–S3X Table and S4A Table–S4X Table).

Conservation scores were allocated following the logic outlined in Fig 1A and S1 Appendix. Due to the difficulties inherent in deriving accurate estimates for numbers of animals held in the majority of WTAs, we allocated scores based on the IUCN Redlist status for the subject taxa. We assumed that negative conservation impacts (e.g. removal of individuals from the wild, transmission of disease) on populations at risk (vulnerable, near threatened, endangered or critically endangered) would represent a considerable conservation disbenefit whereas for species of least conservation concern, larger numbers of individuals could be affected before a substantial negative population effect occurred. Accordingly negative effects for direct conservation impacts were scored as -1 for least concern (LC) species, -2 for vulnerable or near threatened (VU/NT) species, and -3 for endangered or critically endangered (EN/CR) species. Positive conservation effects were scored as LC+1, VU/NT +2, EN/CR+3.

Welfare scores were allocated following the logic outlined in Fig 1B and S1 Appendix, but in brief represent the degree to which attractions within WTA types fulfilled the 'five freedoms' of captive animals—freedom from hunger and thirst; from discomfort; from pain, injury and disease; to behave normally; from fear and distress [31]—which are routinely used to assess the welfare of captive animals [32]. Our assessment of whether a freedom was met was necessarily aggregated across a number of individual WTAs within each type, and freedoms were judged to be partially met if evidence indicated that some WTAs of this type met the requirement while others did not. In the absence of contradictory information or logic each freedom was considered to be fully met by each WTA type. Sanctuary WTAs (which source animals from other captive institutions with the aim of improving their welfare) were assessed as above, but were given positive scores which reflected how many freedoms they improved in comparison with other WTA types involving that taxon (Fig 1B). We assessed WTAs that use non-captive, wild populations by applying the five freedoms principle to the likely impacts of tourists on individuals in those populations. For non-sanctuary WTAs if a WTA type fulfilled all five freedoms it scored 0, four or more freedoms -1, if between one and four freedoms -2 and if one freedom or less -3. These cut-off points were chosen because in practice WTAs with high welfare standards were relatively easy to discern from the supporting literature, as were those with the worst standards, but fine scale separation for the remainder was complicated by variation within WTA types and paucity of information or unreliable sources.

### Collation and analysis of online tourist reviews

To assess tourists’ feedback on the above rated WTA types we used reviews hosted by the internet travel review site TripAdvisor. We were unable to find alternative review sites that covered a sufficient range of relevant WTAs, or respondents from a diversity of countries, and which could provide internally comparable reviews across different WTA types and taxa.

TripAdvisor users score attractions on a five point scale (Terrible = 1, Poor = 2, Average = 3, Very Good = 4, Excellent = 5). Given time constraints and the thousands of relevant reviews, we assumed that scores of Very Good or Excellent were intended by the reviewer to recommend the attraction to other users and for this reason these positive reviews were not read, but used as provided by TripAdvisor. We assumed that reviews scored as Terrible or Poor were intended to deter other users and so classed these as negative. However, it was necessary to distinguish negative reviews that derived from tourists' appreciation of the conservation and welfare impacts of the attraction from those stemming from other negatives (e.g. bad refreshments, rude staff, overcrowding, safety issues etc.). We therefore counted only those negative reviews that specifically contained phrases indicating an awareness of the relevant issues (e.g. “Those poor animals…”, “…great for tourists, bad for elephants…”, “Conservation or exploitation of animals?”, “it [was] really sad and I hate that we gave money to support it”). When reviews were posted in a language other than English, the review was translated through the feature available on TripAdvisor and then assessed as above.

Average reviews, by definition, were neither positive nor negative even when mentioning welfare or conservation concerns (e.g. “…enjoyable day but couldn't help feeling a bit sorry for the animals”). It was often unclear to what extent reviewers who gave this score counted conservation or welfare concerns as a negative factor, and to avoid misrepresenting users' intentions we discounted ‘average’ reviews from our analysis.

Our metric for tourist dissatisfaction (‘tourist dissatisfaction score’), regarding the welfare and conservation conditions of a given WTA, was calculated as the percentage of all reviews—discounting ‘average’ reviews—accounted for by negative reviews (i.e. 100 x (negatives / (positives + negatives))), where a low percentage indicates that few tourists had a negative perception of the attraction.

### Separation of sanctuaries from captive WTAs

WTAs reviewed on TripAdvisor were assigned to WTA types (e.g. wild dolphin interactions, elephant sanctuary; S1 and S2 Tables) to permit comparison with the independent welfare and conservation scores. Difficulties arose when separating sanctuaries from captive WTAs with the same subject taxa because both categories of WTA type maintain individuals in captivity but differ in the intention of the WTA (Fig 1B; S1 Table); attractions in both WTA types, however, often claim welfare credentials in their promotional material. These WTA types could not be separated on the basis of TripAdvisor reviews (i.e. assuming that WTAs with good customer reviews were sanctuaries) because this would predetermine the conclusion that consumers gave better ratings to sanctuaries than comparable WTA types. We therefore separated these WTA types first on the basis of whether a given WTA self-defined as a sanctuary, and then by inspecting the WTA’s mission statement (typically hosted on their website) to determine the manner in which tourists were permitted to interact with the captive animals: we defined elephant sanctuaries as those that did not permit tourists to ride elephants or attend shows, lion sanctuaries as not permitting handling of cubs, and orang-utan and bear sanctuaries as not permitting shows involving these animals. Dolphin sanctuaries presented difficulties in that all dolphin WTAs permitted dolphin interactions, but dolphin sanctuaries differ in actively rescuing, rehabilitating and releasing injured marine animals under license. If in advance of analysing the tourist dissatisfaction scores it remained uncertain which WTA type a particular attraction belonged to, that attraction was removed from further analysis (three individual WTAs were discounted on this basis). We also recorded whether each non-sanctuary WTA claimed to provide conservation or welfare benefits for their subject animals in their promotional material; but because WTAs regularly conflate conservation and welfare we were unable to separate the types of benefit claimed.

### Statistical analysis

We wished to test whether tourist dissatisfaction scores (percentage of negative reviews) correlated with our independently allocated conservation and welfare scores. Welfare and conservation scores represent estimates across all attractions within a WTA type (i.e. the mean expected impact per WTA type) and so we constructed general linear models—implemented in Minitab 15—with mean tourist dissatisfaction score for each WTA type (n = 15) as the response variable and welfare and conservation scores entered as covariates.

In a separate general linear model we tested whether tourist dissatisfaction scores responded to the identity of the subject taxon, whether a given WTA was a sanctuary, comprised wild or captive animals, and whether the WTA claimed to provide conservation and welfare benefits for the subject animals. For both analyses, WTAs with fewer than 30 reviews were discounted to prevent biased percentages resulting from small sample sizes, and percentages were arcsine square-root transformed to meet the assumptions of the test.

## Results

We identified 48 different types of non-zoo WTAs globally (S2 Table), falling within six categories (S1 Table). We selected a shortlist of 24 WTA types from within these categories for in-depth study of their conservation and welfare impacts, and tourists’ satisfaction with attractions within that WTA type (Table 1). These 24 WTA types represented at least 406 individual WTAs worldwide (each rated on TripAdvisor), involving 236,000–561,000 individual animals and catering for 3.5–6 million tourists annually (Table 1; S3A Table–S3X Table and S4A Table–S4X Table). Approximately 100,000 subject animals were not captive, but used for wild tourism (Table 1). Of the remaining 136,000–461,000 captive animals, the greatest numbers in given WTA types were farmed crocodiles (>100,000), farmed sea turtles and captive elephants (both 10,000–100,000), the remainder all representing a maximum of 1,000–10,000 individuals per attraction (Table 1; S3A Table–S3X Table and S4A Table–S4X Table).

### Conservation and welfare scores

**Fig 2:**
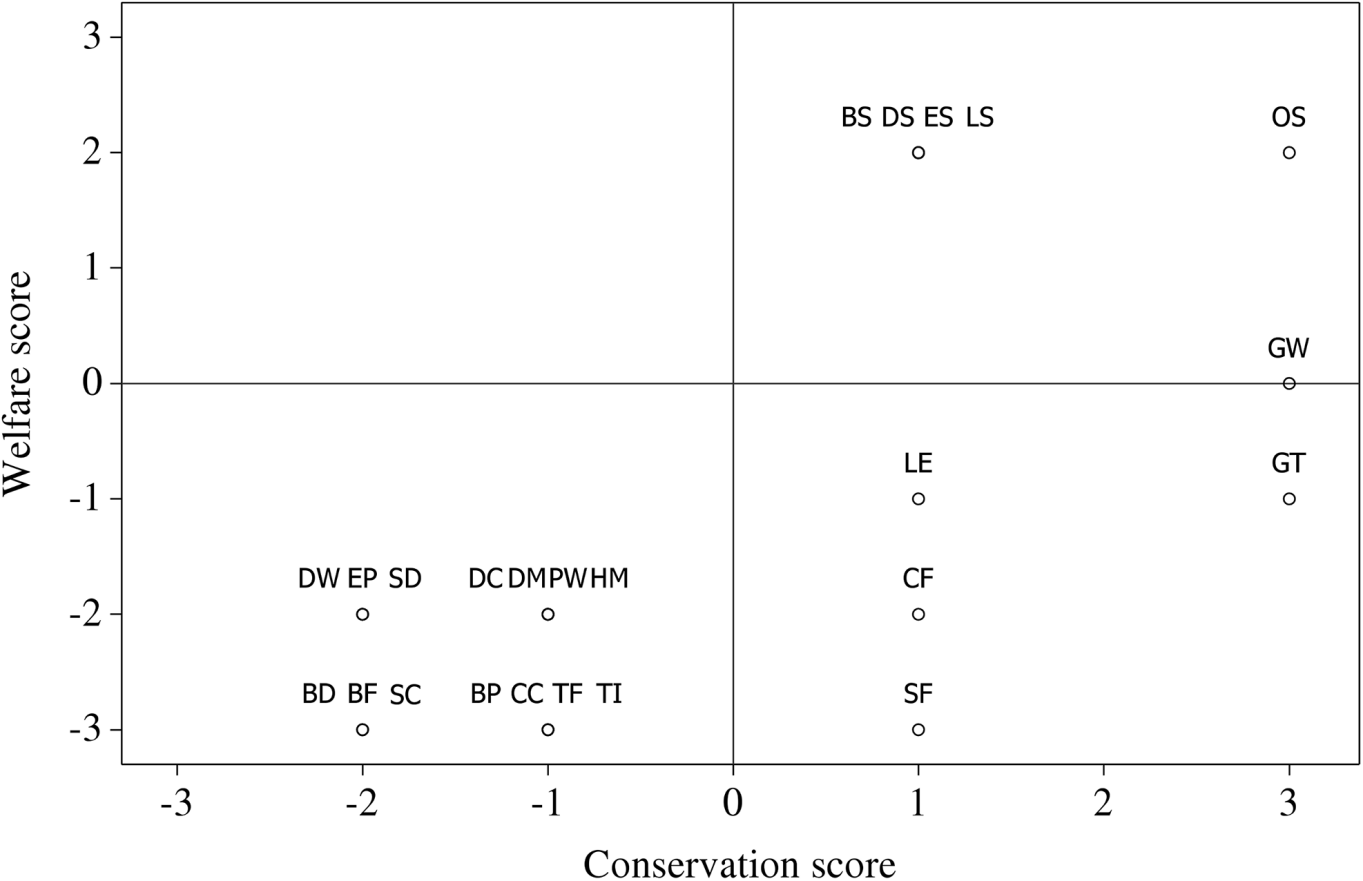
Welfare and conservation scores for the 24 selected WTA types. **BD** = Bear dancing, **BF** = Bear bile farms, **BP** = Bear parks, **BS** = Bear sanctuary, **CC** = Civet coffee, **CF** = Crocodile farms, **DC** = Captive dolphin interactions, **DM** = Dancing macaques, **DS** = Dolphin sanctuary, **DW** = Wild dolphin interactions, **EP** = Elephant parks, **ES** = Elephant sanctuary, **GT** = Gorilla trekking, **GW** = Gibbon watching, **HM** = Hyena men (Nigeria), L**E** = Lion encounters, **LS** = Lion sanctuary, **OS** = Orang-utan sanctuary, **PW** = Polar bear watching, **SC** = Snake charming, **SD** = Shark cage diving, **SF** = Sea turtle farm, **TF** = Tiger farms, **TI** = Tiger interactions.

Of our 24 selected WTA types, only five had positive scores for both the conservation and welfare impacts on the subject taxa and individuals, and all five were sanctuaries (WTAs that source animals from other captive institutions with the aim of improving their welfare and/or conservation status; Table 1; Fig 2; S3A Table–S3X Table and S4A Table–S4X Table). Of the remaining 19 WTA types, five had positive scores for the subject animals’ conservation status (gorilla trekking, gibbon watching, sea turtle farming, crocodile farms, lion encounters), but four of these had negative welfare scores (gorilla trekking, sea turtle farms, crocodile farms, lion encounters), and the remaining 14 had negative scores for both conservation and welfare (Table 1; Fig 2). Of 24 WTA types, therefore, six (five sanctuaries and one wild WTA type, gibbon watching) had net positive impacts, four (gorilla trekking, sea turtle farms, lion encounters, crocodile farms) had positive conservation impacts offset by negative welfare impacts, and 14 had net negative impacts of varying severity. Eighteen WTAs had at least some negative impact on their subject animals (Table 1; Fig 2).

### Tourist dissatisfaction scores

**Fig 3:**
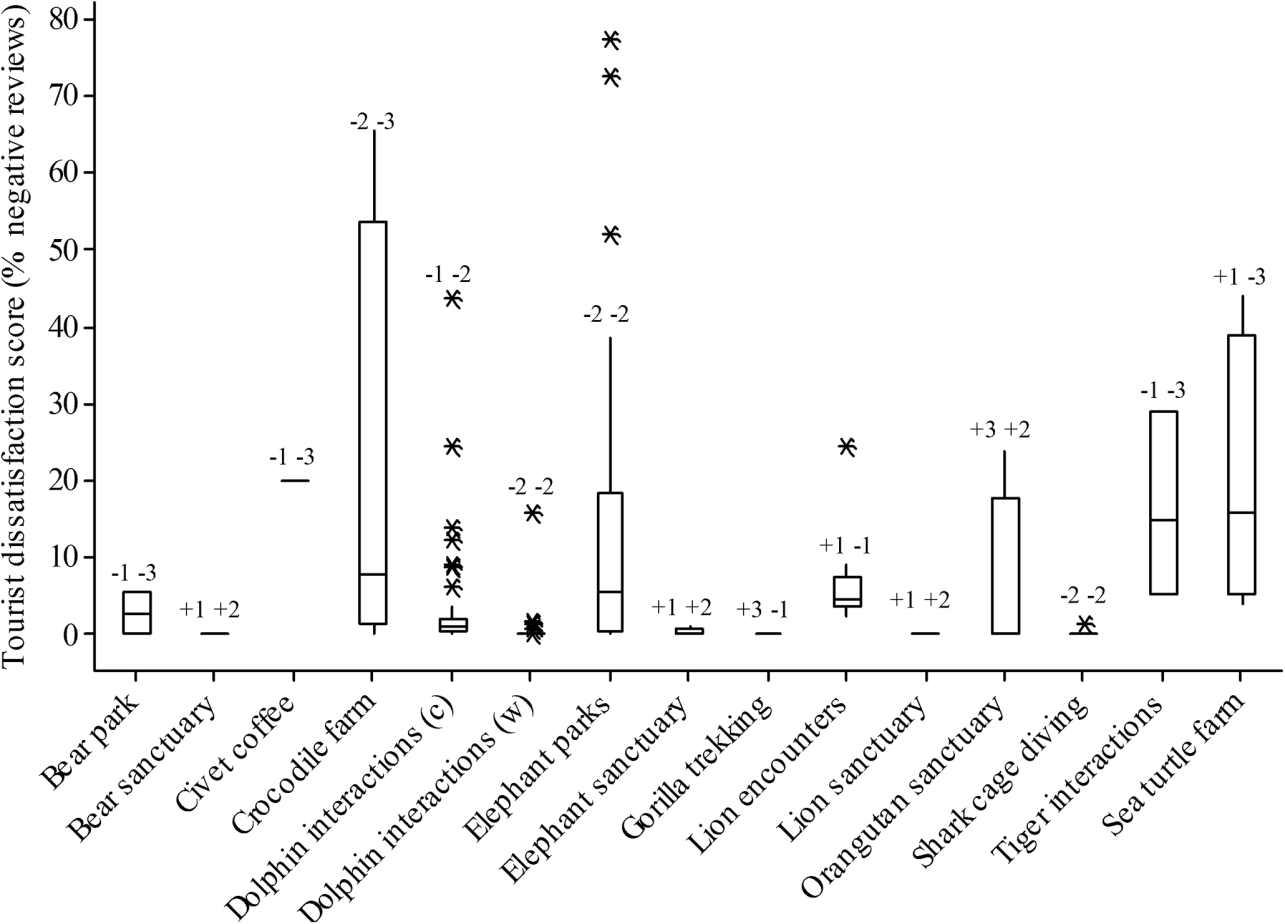
Tourist dissatisfaction scores from TripAdvisor reviews (measured as the percentage of all positive and negative reviews that were negative). Bars represent the median, boxes the interquartile range, and asterisks outlying points. Numbers above each column, for reference, show the independently awarded conservation and welfare scores, respectively, for each attraction. “C” and “W” denote captive and wild dolphin interactions.

We derived tourist dissatisfaction scores for 188 separate WTAs on TripAdvisor, representing 15 of our 24 selected WTA types. Data were either unavailable or insufficient (fewer than 30 reviews) to assess attractions in the remaining nine selected WTA types. Of 51,308 separate reviews, 46,688 were positive and 4,620 were negative, of which 2,439 were negative specifically due to welfare and/or conservation concerns. The mean tourist dissatisfaction score was 7.8% (SD 14.7) across all WTAs. Although some WTA types represented attractions between which tourist dissatisfaction scores varied widely—particularly elephant parks and crocodile farms (Fig 3)—80% of all WTAs had tourist dissatisfaction scores of ≤10%, and 92% of WTAs had scores of ≤ 30%. Only 15 of 188 WTAs had scores of above 30% (range 30.8% to 77.6%) comprising eight elephant parks, five crocodile farms, one captive dolphin interaction and one turtle farm.

Mean tourist dissatisfaction scores for each WTA type (Table 1) negatively correlated with our independently attributed welfare scores (F_1,14_ = 6.94, p = 0.022) in a linear model also containing effects of conservation scores, such that mean tourist dissatisfaction scores ranged from 20.5% for welfare scores of -3, to 1.9% for welfare scores of +2 (no WTA type had a score of +3). There was no evidence for an effect of conservation score on tourist dissatisfaction score (F_1,14_ = 0.37, p > 0.5).

Tourist dissatisfaction scores varied between subject taxa (F_10,187_ = 4.36, p < 0.001; Fig 3), and were lower for sanctuary than non-sanctuary WTAs (F_1,187_ = 7.85, p = 0.006, means 1.9% and 8.3%, respectively), WTAs with wild, as opposed to captive animals (F_1,187_ 4.46 p = 0.36; 0.6% and 9.7%, respectively), and for WTAs that claimed welfare or conservation benefits in their publicity material (F_1,187_ = 4.40, p = 0.37; 5.9% and 9.6%, respectively) in a linear model containing only these explanatory variables. Tukey post-hoc tests for effects of subject taxon revealed that dolphins had significantly lower mean tourist dissatisfaction scores than crocodiles and elephants (T = 5.090, p < 0.001; T = 4.1062, p < 0.05, respectively) and that sharks had lower scores than crocodiles (T = 5.887, p < 0.001), all other comparisons being non-significant (T < 3.2, p > 0.05 in all cases; c.f. Fig 3). The total number of all negative TripAdvisor reviews for each WTA was highly correlated with the number given explicitly for welfare and/or conservation reasons (Pearson correlation = 0.923, p <0.001).

## Discussion

Of the 24 WTA types we analysed 14 had negative impacts on both the welfare and conservation status of their subject taxa, 18 WTA types had negative welfare impacts and six had positive impacts overall (Fig 2). Attractions in our list of 24 WTA types collectively negatively impacted the welfare status of 230,000–550,000 individual animals (Table 1) and 120,000–340,000 animals were employed in WTAs that reduced the conservation status of their wild populations. By comparison, only 1500–13,000 individuals were employed in attractions (five sanctuary attractions, and one wild attraction; Table 1) that contributed positively (or were neutral) to both welfare and conservation. A further 100,000 individuals were in crocodile farms (with overall conservation benefits, but negative welfare impacts). Overall at least 2–4 million of the 3.6–6 million tourists per annum visiting attractions within these 24 WTA types supported, through patronage, institutions that contribute to negative welfare and/or conservation impacts. Due to limitations of time and data availability we were unable to audit the full list of 48 WTA types we identified worldwide (S2 Table), but–while acknowledging the many uncertainties concerning the impacts of, and numbers of animals and tourists involved in, the remaining WTA types–it seems reasonable to expect the above figures to be substantially increased, and potentially more than doubled, if a full global audit of every WTA were possible.

Mean tourist dissatisfaction score inversely correlated with welfare scores, such that the percentage of negative tourist reviews decreased from approximately 17% to 6% as welfare scores increased from -3 to +2. Overall, however, our figures indicate that the majority of attending tourists did not recognise and/or respond to negative welfare impacts: typically 80% did not. For the 15 WTA types—comprising 188 individual WTAs—for which sufficient data were available only 7.8% of all tourist feedback was negative due to conservation or welfare concerns, and for the eight WTA types (comprising 24 individual WTAs) to which we allocated the lowest welfare scores of -3, the mean tourist dissatisfaction score was 20.5%. As an example, of 3904 reviews of tiger interaction attractions (n = 3, welfare score = -3), 3205 (82%) reviews rated the attraction as “Excellent” or “Very Good” and only 699 (18%) were negative due to welfare/conservation considerations.

A minority of WTAs (8%, 15 of the 188 analysed) had tourist dissatisfaction scores exceeding 30%, and only two (both elephant parks) had tourist dissatisfaction scores exceeding 70% (for these 62 of 82 reviews were negative; example review: “I have never been so upset. Those poor animals. Something really needs to be done. Its [sic] cruel, nasty and the filth these poor helpless animals are living in…”[33]). In these cases we speculate that tourists responded to particularly conspicuous welfare abuses, potentially indicating that less obviously compromised welfare standards in other WTAs remained undetected by the majority of tourists. Similarly, impacts may be more difficult to detect in some taxa than others [34]. Captive dolphin interactions and elephant park WTA types were both allocated the same welfare score (-2), but mean tourist dissatisfaction scores for elephant parks were significantly higher than for captive dolphin interactions (12.5%, SD 17.8, n = 55, and 3.5%, SD 7.6, n = 46, respectively). We cannot discern if this discrepancy stems from the demographic composition of, and expectations of, the clientele (e.g. tourists motivated to experience a “bucket list” activity, may be less concerned with, or likely to detect, negative welfare impacts on subject animals [3]), or from another internal bias (e.g. it may be less easy to judge welfare conditions relatively unfamiliar marine environments) but overall our results imply that tourists have an imperfect perception of the welfare consequences of WTAs. Education of tourists in these consequences could decrease tourist demand and/or drive improvements in the standards of WTAs [35]. No association between conservation score and tourist dissatisfaction score was detected, potentially because conservation impacts are less likely to be visible as part of tourists’ immediate experience of a given attraction.

Reviews on TripAdvisor are not a random sample and may not be fully independent because reviewers self-select and are potentially influenced by other reviews. Also if tourists decide *a priori* not to attend a given attraction, potentially on ethical grounds, they may not leave reviews. For these reasons caution is required in drawing conclusions about the absolute percentages of tourists who perceived or responded to the welfare conditions at WTAs—to accurately derive such figures would require questionnaire surveys conducted *in situ*. However, TripAdvisor is the largest global internet review website [29], providing top lists of attractions in various locations. Positive ratings for a given WTA will encourage further tourists to visit while negative ratings will discourage visits. Tourist dissatisfaction scores are a measure of the degree to which WTAs are recommended to future visitors, and indicate whether tourists’ perception of welfare standards at a WTA could limit numbers of future visitors.

Given the few peer-reviewed studies and NGO reports on, and relatively recent emergence of, the majority of the WTAs, inaccuracies in the welfare and conservation scores we awarded are inevitable. However the logic by which the scores were allocated was structured to avoid systematic overestimate of the severity of negative impacts (Fig 1A and 1B), and where information was lacking or equivocal WTAs were assumed to have no negative effect. While the precise score for a given attraction may alter if new information were available the underlying logic by which scores were assigned would prevent the direction of an impact (positive or negative) from changing for the majority of WTA types. The only potential exceptions were conservation scores for lion encounters and sea turtle farming, which were +1 on the basis of their provision of conservation education (Table 1; Fig 1A). The conservation approach of both WTA types has been criticised in the scientific literature [25, 26], however, and if the education they provide were shown to promote inappropriate conservation activities it may then be judged to be of equivocal value (scoring 0) or counter-productive (-1).

Auditing the full global diversity of wildlife tourist attractions and their impacts in this study would have been impractical, and there undoubtedly exist WTA types not listed in S2 Table. Our study focussed only on animals, in particular on those for which types of WTAs could be identified and for which sufficient information was available to accurately assess the conservation and welfare consequences for the species in question. WTAs may have welfare and conservation impacts beyond their focal species but we did not consider these in this study. Our findings suggest a mammal-bias: 20 of the 24 WTA types in Table 1 had mammals as their focus, as did 32 of the 48 WTA types in the full list (S2 Table). Despite extant WTAs which have as their focus species of reptiles, birds or invertebrates (see S2 Table), there appears to be a broad preference for the use of mammal species by WTA operators, particularly in captive and sanctuary attractions (S2 Table; Table 1).

In summary this paper comprises the first attempt to audit the conservation and welfare impacts of the array of non-consumptive, non-zoo wildlife tourist attractions now available worldwide. We conclude—by extrapolating from the results of the subset of 24 attractions we analysed in detail—that the majority (approximately two thirds to three quarters) of wildlife tourist attractions have negative welfare impacts on individual animals and on their taxon’s conservation status. A small proportion of tourists attending a given WTA recognise and respond to the welfare status of the subject animals, but typically 80% or more will not.

Two recommendations arise from this work. First, given the substantial disagreement between our objective assessment and the tourist’s subjective reviews, and the large numbers of tourists attending attractions with poor welfare standards, tourist feedback appears insufficient to regulate the use of animals in WTAs. The large number of animals and tourists involved, as well as the predicted future increases in global tourism, indicate an urgent need for regulation, in the form of an accreditation or certification schemes [36, 37], policy instruments (e.g. taxation or quota fixing; [38] or agencies to inspect and sanction WTAs globally. Second, tourist education in the effects of their patronage of WTAs is necessary. Such education could effectively be provided by professional writers (typically via hard copy travel guides), and through online consumer reviews. In particular the most accessible and popular source of online feedback on WTAs worldwide is TripAdvisor, which provides a prominently placed statistic for each attraction, detailing the percentage of positive reviews overall. Given the strong correlation between total negative reviews and those arising from conservation/welfare concerns, and the large proportion of positive reviews awarded even to attractions with severe welfare impacts, we suggest that WTAs for which the TripAdvisor score is 80% or less are likely to indicate those that have negative animal welfare consequences. Tourists not wishing to participate in attractions which foster welfare abuses or hamper conservation might use this suggestion as a rough indication of suitability.

## Supporting Information

S1 Appendix Supplementary methods. Detailed rationale for the allocation of conservation and welfare scores, to accompany Fig 1A and Fig 1B.
(DOCX)

S2 Appendix Supplementary references cited. Additional references cited, S2, S3 and S4 Tables (see relevant tables in Supporting Information).
(DOCX)

S1 Data TripAdvisor analysis data deposit. Table showing the name, WTA (wildlife tourist attraction) type, WTA category, geographical location, URL and the feedback ratings left on TripAdvisor, for the 188 wildlife tourist attractions assessed for the creation of tourist dissatisfaction scores. Figures in columns “Terrible” to “Excellent” refer to the number of tourists who elected this response to the WTA on TripAdvisor on the date the WTA was assessed. The following two columns detail how many ‘Terrible’ and ‘Poor’ reviews for a given WTA were discounted due to being awarded for reasons other than welfare or conservation standards. Columns “Positive” and “Negative” contain the overall number of positive reviews, and of negative reviews awarded on the basis of conservation and welfare standards. The column “Percentage” contains the tourist dissatisfaction score.
(XLSX)

S1 Table Categorisation of types of wildlife tourist attractions. (DOCX)

S2 Table Full list and description of all types of wildlife tourist attraction currently operating that were discovered during the desk study. See S2 Appendix for reference citations.
(DOCX)

S3 Table Detailed justifications and sources underpinning the allocation of conservation scores for the 24 wildlife attraction types (WTA types) selected for in-depth study (see Table 1; see Fig 1A and S1 Appendix Methods for underlying logical structure), the derivation of numbers of tourist and animals involved (Table 1), and separation of sanctuary and non-sanctuary attractions. WTA types are listed alphabetically. See S2 Appendix for reference citations.
(DOCX)

S4 Table Detailed justifications and sources underpinning the allocation of welfare scores for the 24 wildlife attraction types (WTA types) selected for in-depth study (shown in Table 1; see Fig 1B and S1 Appendix for underlying logical structure). Scores are based on the degree to which the five freedoms of captive animals are fulfilled. A score of 1 indicates that a give freedom was likely to be fulfilled by all WTAs within a given WTA type; conversely a score of 0 indicates that all WTAs were unlikely to fulfil the freedom. A score of <1 indicates that WTAs within a given type may vary in the extent to which they meet this freedom. WTA types are listed alphabetically. See S2 Appendix for reference citations.
(DOCX)
