# Supplementary material for: The Customer Isn't Always Right—Conservation and Animal Welfare Implications of the Increasing Demand for Wildlife Tourism
Source: PLoS One. 2015 Oct 21;10(10):e0138939. doi: 10.1371/journal.pone.0138939 (PMC4619427; doi:10.1371/journal.pone.0138939)
Supplement: S1 Appendix — Detailed rationale for the allocation of conservation and welfare scores, to accompany Fig 1A and Fig 1B. (DOCX) [file pone.0138939.s001.docx]

**S1 Appendix**

**Detailed Rationale of Conservation Scores**

The below decision tree accompanies Fig. 1a, providing further details and justifications for each available choice.

*Conservation scores Q1: Are animals captive or wild?*

For captive, see Q2. For wild, see Q5.

*Conservation scores Q2: For captive animals, what is the source of animals?*

If animals in a captive attraction were principally sourced from the wild, the attraction was considered to have an overall negative impact, in the absence of evidence of a countervailing conservation benefit - see Q3. If captive bred see Q4.

Conservation scores Q3: *For captive animals, wild sourced, is the conservation impact of the removal of individuals from wild populations offset by an overall conservation benefit?*

If no countervailing benefit existed, the degree of the negative impact of the removal of individuals was related to the IUCN Redlist conservation status of the wild population of the taxon/taxa in question (least concern, LC, -1; vulnerable or near threatened, VU/NT, -2; endangered or critically endangered, EN/CR, -3). It is possible that WTAs that capture animals from the wild may nonetheless participate in conservation-relevant activities (e.g. tourist education, or provision of funds for protected areas), and may potentially provide an overall conservation benefit. Accordingly we allowed for the allocation of positive scores (LC+1, VU/NT +2, EN/CR+3) when clear evidence existed of an overall benefit, and of a neutral score where it was plausible that the combined removal of individuals and conservation activities may counteract. (In practice, however, no such trade-offs were found among the WTAs reviewed.)

*Conservation scores Q4: For captive animals, captive bred*, *is there evidence of a direct conservation benefit?* Animals bred in captivity were assumed to have no direct negative impact on wild populations. If evidence existed that the attraction has directly benefited the subject species' conservation (e.g. through successful reintroduction, or through the provision of funds to secure habitats), then a positive score was awarded (LC+1, VU/NT +2, EN/CR+3). If no direct benefit, see below.

*Conservation scores Q5: For captive animals, captive bred, no direct conservation benefit, does the attraction provide conservation relevant education?* In the absence of a direct conservation benefit, if the attraction educates visitors in conservation we assume that it will have an overall positive effect (+1; sensu [1, 2]), but if no such education existed then we assumed a negative effect (-1), due to the implicit promotion of the acceptability of entertainment-only wildlife exhibits [3-6]. In some cases attractions could provide conservation education which may contain a proportion of incorrect or mis- information, and therefore both beneficial and detrimental aspects. In such equivocal cases a score of 0 was awarded.

*Conservation scores Q6: For wild animals, is there evidence of direct conservation benefit?*

Positive scores were attributed to those attractions with evidence of direct conservation benefits (e.g. anti-poaching action, provision of funds for habitat management/ purchase) for those populations / species, allocated in relation to the IUCN Redlist status for that species (LC+1, VU/NT+2, EN/CR+3). If no direct benefit, see below.

*Conservation scores Q7: For wild animals, no direct conservation benefit, is there evidence of a direct conservation disbenefit?* If the attraction had a recorded negative impact (e.g. though increasing disease exposure, or disturbance likely to have survival consequences) negative points were allocated (LC -1, VU/NT -2, EN/CR -3). If no direct negative impact, see below.

*Conservation scores Q8: For wild animals, no direct conservation benefit or disbenefit, does the attraction provide conservation relevant education?*

The attraction was allocated +1 if it educated visitors in conservation, -1 if not and 0 if the case was equivocal, following logic in Q3 above.

**Detailed Rationale for Welfare Score allocation**

The below decision tree accompanies Fig. 1b, providing further details and justifications.

*Welfare scores Q1: Are animals captive or wild?*

*For wild, see Q2. For captive, see Q3.*

Welfare scores Q2: For wild animals, if the attraction fulfilled all of the five freedoms (i.e. no welfare consequences) it was given a score of 0. Otherwise attractions scored -1 if fulfilling four to five freedoms, -2 if fulfilling at least one, but fewer than four, freedoms and -3 if fulfilling zero or one freedom. For captive animals see below.

*Welfare scores Q3: For captive animals, does attraction primarily exist to improve the net welfare of the subject animals (i.e. a 'sanctuary', sourcing animals from other captive institutions with the intention of providing better conditions) or as a draw for tourist revenue (an 'attraction')?*

For sanctuaries we allocated +1 if the sanctuary improved at least one freedom for the subject animal (in comparison with other attractions using that species), +2 if it improved more than two, but fewer than five freedoms, +3 if all five freedoms were improved. If no freedoms were improved then the attraction was not a sanctuary, see Q3.

*Welfare scores Q4: Animals in attraction, where the net animal welfare was not improved.*

Attractions scored -1 if fulfilling four or more freedoms, -2 if fulfilling between one and four freedoms, and -3 if fulfilling one freedom or less.
