# Supplementary material for: The Customer Isn't Always Right—Conservation and Animal Welfare Implications of the Increasing Demand for Wildlife Tourism
Source: PLoS One. 2015 Oct 21;10(10):e0138939. doi: 10.1371/journal.pone.0138939 (PMC4619427; doi:10.1371/journal.pone.0138939)
Supplement: S2 Appendix — Additional references cited, S2, S3 and S4 Tables (see relevant tables in Supporting Information). (DOCX) [file pone.0138939.s002.docx]

**S2 Appendix**

1. Ballantyne R, Packer J, Sutherland LA. Visitors’ memories of wildlife tourism: Implications for the design of powerful interpretive experiences. Tourism Management. 2011;32(4):770-9.

2. Zeppel H, Muloin S. Conservation benefits of interpretation on marine wildlife tours. Human Dimensions of Wildlife. 2008;13(4):280-94.

3. Fien J, Scott W, Tilbury D. Education and Conservation: Lessons from an evaluation. Environmental Education Research. 2010;7(4):379-95.

4. Lück M. Education on marine mammal tours as agent for conservation—but do tourists want to be educated? Ocean & Coastal Management. 2003;46(9):943-56.

5. Orams MB. The effectiveness of environmental education: can we turn tourists into 'Greenies'? Progress in tourism and hospitality research. 1997;3:295-306.

6. Zeppel H. Education and conservation benefits of marine wildlife tours: Developing free-choice learning experiences. The Journal of Environmental Education. 2008;39(3):3-18.

7. Videoportal3000. Circus Elephants perform: YouTube.com; 2011 [11/01/2015]. Available from: <https://www.youtube.com/watch?v=NSo1Gp1bd7Y>.

8. World Animal Protection. Ending bear baiting [12/01/2015]. Available from: <http://www.worldanimalprotection.org/our-work/animals-wild/ending-bear-baiting>.

9. Education for Nature - Vietnam. Bear bile tourism finally shut down in Ha Long 2014 [23/12/2014]. Available from: <http://envietnam.org/index.php/topic/env-activities/638-bear-bile-tourism-finally-shut-down-in-ha-long>.

10. Advocacy for Animals. The Dancing Bears of India: Moving Toward Freedom 2011 [18/11/2014]. Available from: <http://advocacy.britannica.com/blog/advocacy/2007/12/the-dancing-bears-of-india-moving-toward-freedom/>.

11. JapanGuides.net. Noboribetsu Bear Park [14/11/2014]. Available from: <http://www.japanguides.net/hokkaido/noboribetsu-bear-park.html>.

12. Bear Sanctuary. Libearty Bear Sanctuary [15/12/2014]. Available from: <http://bearsanctuary.com/libearty-bear-sanctuary>.

13. The Sun Bear Education and Conservation Centre. Sun bear enclosure [31/12/2014]. Available from: <http://en.kwplh.beruangmadu.org/educational-facilities/sun-bear-enclosure/>.

14. Naturetrek. Birds 2015 [20/08/2015]. Available from: <http://www.naturetrek.co.uk/landingpage.aspx?url=bird-watching-holidays#.VdW3hpdLaew>.

15. Sunbird Tours. 2015 [20/08/2015]. Available from: <http://www.sunbirdtours.co.uk/>.

16. Dalat Trip. Da Lat Weasel Coffee – Civet Coffee’s real flavor [24/12/2014]. Available from: <http://www.dalattrip.com/trai-ham-da-lat-weasel-coffee-civet-coffees-real-flavor/?shared=email&msg=fail>.

17. PADI. The Great Barrier Reef 2015 [20/08/2015]. Available from: <http://www.padi.com/scuba-diving/scuba-diving-travel/vacation-spotlights/great-barrier-reef/>.

18. Worldcrocodile.com. Samutprakan crocodile farm and zoo [25/12/2014]. Available from: <http://www.worldcrocodile.com/souvenir.html>.

19. Malta Marine Park. Swim with dolphins [18/12/2014]. Available from: <http://www.marineparkmalta.com/dolphins>.

20. Dolphin Discovery. Dolphin swim adventure in Cozumel [18/12/2014]. Available from: <http://www.dolphindiscovery.com/cozumel/cozumel-activities-dolphin-swim-adventure.asp>.

21. Dolphin Swims Wild and Free. Onboard adventure dolphin safari [22/01/2015]. Available from: <http://www.dolphinswims.co.uk/dolphin-swim-holidays/onboard_adventure/>.

22. Dolphin Encounter. Welcome to Dolphin Encounter Kaikoura [21/01/2015]. Available from: <http://www.dolphinencounter.co.nz/;>.

23. Dolphin Research Centre. Plan your day [01/01/2015]. Available from: <http://www.dolphins.org/plan_your_day?aid=1>.

24. Ecoventures. Wildlife watch [23/01/2015]. Available from: <http://www.ecoventures.co.uk/trips/wildlife-watch/>.

25. Knysna Elephant Park. Elephant rides [18/12/2014]. Available from: <http://www.knysnaelephantpark.co.za/experiences/rides/>.

26. Kok Chang Safari. Welcome to Kok Chang Safari elephant trekking, Phuket [18/12/2014]. Available from: <http://www.kokchangsafari.com/;>.

27. Dude in Bali. Bakas elephant tour [18/12/2014]. Available from: <http://www.dudeinbali.com/activities/bakas-elephant-tour/>.

28. Elephant Nature Park. Pamper a Pachyderm - Full Day Visit [02/01/2015]. Available from: <http://www.elephantnaturepark.org/enp/en/visit-volunteer/projects/viewproperty/pamper-a-pachyderm-full-day-visit/44?thisProperty=44>.

29. Tong Bai Elephant Tour. Tour [15/12/2014]. Available from: <http://tongbai-elephant-tour.com/?page_id=43>.

30. Wilson S. New Zealand fur seal: Seal Conservation Society; [24/01/2015]. Available from: <http://www.pinnipeds.org/seal-information/species-information-pages/sea-lions-and-fur-seals/new-zealand-fur-seal>.

31. Gannet Safaris. 2015 [20/08/2015]. Available from: <http://www.gannetsafaris.co.nz/>.

32. Gibbon Spotting Cambodia. Cambodia's first ever gibbon experience [15/12/2014]. Available from: <http://www.gibbonspottingcambodia.com/app/>.

33. On the Go Tours. Gorillas in the mist [26/01/2015]. Available from: <http://www.onthegotours.com/Africa/Overland-Safaris-East-Africa/Gorillas-in-the-Mist;>.

34. Bwindi National Park. Gorilla trekking in Uganda, Bwindi Forest, gorilla safari reviews [15/12/2014]. Available from: <http://www.bwindiforestnationalpark.com/gorilla-trekking-uganda-bwindi-safari.html>.

35. So Bad So Good. A Fascinating Look At The Hyena Men Of Nigeria [17/12/2014]. Available from: <http://sobadsogood.com/2012/05/23/a-fascinating-look-at-the-hyena-men-of-nigeria-by-photographer-pieter-hugo/>.

36. CCTV.com. Face of Africa: The hyena men: YouTube; 2013 [19/01/2015]. Available from: <https://www.youtube.com/watch?v=fM3BuQneSk4>.

37. Okarito Kiwi Tours. 2015 [20/08/2015]. Available from: <http://www.okaritokiwitours.co.nz/>.

38. Safari Adventures Mauritius. Interactions with lions [18/12/2014]. Available from: <http://www.safari-adventures-mauritius.com/lions-cheetahs-tigers/files/page.php?id=22&lang=1>.

39. Lion Encounter. Lion interaction [19/12/2014]. Available from: <http://www.lionencounter.com/activities/lion-interaction>.

40. Zambezi Safaris. Lion encounters. Available from: <http://www.victoriafalls.net/explore-victoria-falls/victoria-falls/lions.aspx#.VMIjiy5H50Y>.

41. Drakenstein Lion Park. [04/01/2015]. Available from: <http://www.lionrescue.org.za/visitorinfo.htm>.

42. Lions Tigers and Bears. Animal encounters [23/01/2015]. Available from: <http://lionstigersandbears.org/visit-us/animal-encounters>.

43. Melborn and Victoria Information. 2015 [18/08/2015]. Available from: <http://www.visitvictoria.com/regions/Phillip-Island/Things-to-do/Nature-and-wildlife/Wildlife-and-zoos/Phillip-Island-Nature-Parks.aspx>.

44. Schmidt-Burbach J. Asian elephant (*Elephas maximus*), pig-tailed macaque (*Macaca nemestrina*) and tiger (*Panthera tigris*) populations at tourism venues in Thailand and aspects of their welfare. Submitted.

45. SouthAfrica.net. Meerkat adventures, Oudtshoorn [28/01/2015]. Available from: <http://www.southafrica.net/za/en/articles/entry/article-southafrica.net-meerkat-encounter-in-oudtshoorn>.

46. Visit Mexico. 2015 [18/08/2015]. Available from: <http://www.visitmexico.com/en/michoacan-where-monarch-butterflies-migrate>.

47. Orangutan Appeal UK. Sepilok orangutan rehabilitation centre [17/12/2014]. Available from: <http://www.orangutan-appeal.org.uk/about-us/sepilok-rehabilitation-centre>.

48. Riordan K. An investigation into using wild animals as photographic props in holiday resorts: WildlifeExtra.com; [13/01/2015]. Available from: <http://www.wildlifeextra.com/go/world/wild-animals-as-photographic-props.html#cr>.

49. Viator.com. Hong Kong pink dolphin watching cruise [29/01/2015]. Available from: <http://www.viator.com/tours/Hong-Kong/Hong-Kong-Pink-Dolphin-Watching-Cruise/d14-3675HKDW;>.

50. Hong Kong Asia's World City. Dolphin watching tour [30/01/2015]. Available from: <http://www.discoverhongkong.com/ca/see-do/tours-walks/guided-tours/nature/dolphin-watching.jsp>.

51. Natural Habitat Adventures. Churchill polar bear tours [31/01/2015]. Available from: <http://www.nathab.com/polar-bear-tours/;>.

52. The Tundra Buggy Adventure. View polar bears in the wild [01/02/2015]. Available from: <http://www.frontiersnorth.com/the-tundra-buggy-adventure>.

53. Townsend W. 'Rattlesnake Roundup' teaches cruelty is fun: CNN; 2014 [14/01/2015]. Available from: <http://edition.cnn.com/2014/04/09/opinion/townsend-rattlesnake-roundup/;>.

54. McDermott R, Owen A. On the road with the American festivals project: National Geographic; [15/01/2015]. Available from: <http://video.nationalgeographic.com/video/american-festivals-project/on-the-road?source=relatedvideo>.

55. Singh A. Reindeer dying in Britain because 'climate too warm': The Telegraph; 2014 [21/12/2014]. Available from: <http://www.telegraph.co.uk/news/earth/wildlife/11283517/Reindeers-dying-in-Britain-because-climate-too-warm.html>.

56. Khama Rhino Sanctuary. Activities [07/01/2015]. Available from: <http://www.khamarhinosanctuary.org.bw/activities>.

57. Rainforest Expeditions. Giant otter [02/02/2015]. Available from: <http://www.perunature.com/giant-otter-pteronura-brasiliensis.html>.

58. Royal Albatross Centre. 2015 [20/08/2015]. Available from: <http://albatross.org.nz/otago-peninsula-tours/>.

59. Safari Bookings. 6-Day Tanzania Highlights Safari [04/02/2015]. Available from: <https://www.safaribookings.com/tours/t1634>.

60. Kuoni. Eastern Cape Game Reserves holidays [03/02/2015]. Available from: <http://www.kuoni.co.uk/south-africa/eastern-cape-game-reserves;>.

61. Cayman Turtle Farm. Turtle encounters [26/12/2014]. Available from: <http://www.turtle.ky/turtle-encounters>.

62. White Shark Diving Co. The great white shark capital of the world [06/02/2015]. Available from: <http://www.sharkcagediving.co.za/>.

63. Shark Cage Diving Calypso Star Charters. Shark cage dives [05/02/2015]. Available from: <http://www.sharkcagediving.com.au/shark-tours/;>.

64. Wild Animals Rescue Foundation of Thailand. [08/01/2015]. Available from: <http://www.warthai.org/view.php?id=1>.

65. Wildlife SOS India. [20/01/2015]. Available from: <http://www.wildlifesos.org/rescue/reptiles/snake-charming>.

66. Tour T. Mae sa snake farm [27/12/2014]. Available from: <http://www.thai-tour.com/eng/chiangmai/maesa-snakefarm/;>.

67. Amazing Thailand. Mae Sa snake farm [28/12/2014]. Available from: <http://uk.tourismthailand.org/See-and-Do/Sights-and-Attractions-Detail/Mae-Sa-Snake-Farm--4016>.

68. Malm S. Made to walk on stilts to entertain tourists, the baby monkey forced to perform when it is choked with a chain in Indonesia: The Daily Mail; 2013 [17/01/2015]. Available from: <http://www.dailymail.co.uk/news/article-2263907/Made-walk-stilts-entertain-tourists-baby-monkey-forced-perform-choked-chain-Indonesia.html>.

69. Fullerton J. Save the Tiger: The animals bred for bones on China’s tiger farms The Independent; 2014 [17/12/2014]. Available from: <http://www.independent.co.uk/voices/commentators/save-the-tiger-the-animals-bred-for-bones-on-chinas-tiger-farms-9636537.html>.

70. Thailand Tour Centre. Tiger Temple Thailand [22/12/2014]. Available from: <http://tigertemplethailand.com/about_tiger_temple_thailand.php>.

71. National Tiger Sanctuary. [09/01/2015]. Available from: <http://www.nationaltigersanctuary.org/tours_and_fees/hours.html;>.

72. Tiger Haven. [10/01/2015]. Available from: <http://www.tigerhaven.org/page.asp?p=About%20Tiger%20Haven>.

73. Elding Adventure at Sea. Whale watching tours [07/02/2015]. Available from: <http://elding.is/tours?type=whalewatching>.

74. Feng Y, Siu K, Wang N, Ng K-M, Tsao S-W, Nagamatsu T, et al. Bear bile: dilemma of traditional medicinal use and animal protection. Journal of ethnobiology and ethnomedicine. 2009;5:1-9.

75. Animals Asia. End Bear Bile Farming [13/11/2014]. Available from: <https://www.animalsasia.org/uk/our-work/end-bear-bile-farming>.

76. Joong Anh Daily Vietnamese urge Koreans not to travel for bear bile 2009 [cited 2014 13/11/14]. Available from: <http://envietnam.org/library/Articles%20for%20news%20media%20section/Vietnamese_urge_Koreans_not_to_travel_for_bear_bile.html>.

77. Clutton-Brock TH, Iason GR. Sex ratio variation in mammals. Q Rev Biol. 1986;61(3):339-74.

78. Garshelis DL, Steinmetz R. (IUCN SSC Bear Specialist Group). *Ursus thibetanus*. The IUCN Red List of Threatened Species. Version 2014.3 2008. Available from: <http://www.iucnredlist.org/details/22824/0>.

79. Dutton AJ, Gratwicke B, Hepburn C, Herrera EA, Macdonald DW. Tackling unsustainable wildlife trade. 2013:74-91.

80. Wildlife SOS India. Dancing Bears [18/11/2014]. Available from: <http://www.wildlifesos.org/rescue/bears/dancing-bears>.

81. D'Cruze N, Sarma UK, Mookerjee A, Singh B, Louis J, Mahapatra RP, et al. Dancing bears in India: A sloth bear status report. Ursus. 2011;22(2):99-105.

82. Seshamani G, Satyanarayan K. The dancing bears of India. World Society for the Protection of Animals. 1997(August).

83. McLellan BN, Servheen C, Huber D. (IUCN SSC Bear Specialist Group) 2008. *Ursus arctos*. The IUCN Red List of Threatened Species. Version 2014.3. [18/11/2014]. Available from: <http://www.iucnredlist.org/details/41688/0>.

84. Garshelis DL, S. R, Chauhan NPS. (IUCN SSC Bear Specialist Group) 2008. Melursus ursinus. The IUCN Red List of Threatened Species. Version 2014.3. [18/11/2014]. Available from: <http://www.iucnredlist.org/details/13143/0>.

85. Wild Welfare. Japanese Bear Parks [14/11/2014]. Available from: <http://www.wildwelfare.org/#!japan/ccma>.

86. Noboribetsu Overview 2014 [18/11/2014]. Available from: <http://www.city.noboribetsu.lg.jp/docs/2013111100017/>.

87. World Animal Protection. Japanese Bear Parks [14/11/2014]. Available from: <http://www.worldanimalprotection.ca/ourwork/bears/japanesebearparks.aspx>.

88. Free the Bears. [21/11/2014]. Available from: <http://www.freethebears.org.au/web/About-Us>.

89. Bear Sanctuary. 2012 [21/11/2014]. Available from: <http://bearsanctuary.com/bear-sanctuaries-around-world>.

90. D'Cruze N, Toole J, Mansell K, Schmidt-Burbach J. What is the true cost of the world's most expensive coffee? Oryx. 2014;48(2):169-71.

91. Wild T. Civet coffee: why it's time to cut the crap 2013 [18/11/2014]. Available from: <http://www.theguardian.com/lifeandstyle/wordofmouth/2013/sep/13/civet-coffee-cut-the-crap>.

92. Widmann P, De Leon J, Duckworth JW. *Arctictis binturong*. The IUCN Red List of Threatened Species. Version 2014.3. 2008 [18/11/2014]. Available from: <http://www.iucnredlist.org/details/41690/0>.

93. Tosun DD. Crocodile Farming and Its Present State in Global Aquaculture. Journal of Fisheries Sciences. 2013;7(200):43-57.

94. Clark JC. A concise history of Florida. Charleston: The History Press; 2014.

95. Crocodile Specialist Group. *Alligator mississippiensis*. The IUCN Red List of Threatened Species. Version 2014.3. 1996 [17/12/2014]. Available from: <http://www.iucnredlist.org/details/46583/0>.

96. Martins M. *Crocodilurus amazonicus*. The IUCN Red List of Threatened Species. Version 2014.3. 2010 [17/12/2014]. Available from: <http://www.iucnredlist.org/details/178328/0>.

97. Choudhury BC, Singh LAK, Rao RJ, Basu D, Sharma RK, Hussain SA, et al. *Gavialis gangeticus*. The IUCN Red List of Threatened Species. Version 2014.3. 2007 [17/12/2014]. Available from: <http://www.iucnredlist.org/details/8966/0>.

98. Crocodile Specialist Group. *Crocodylus intermedius*. The IUCN Red List of Threatened Species. Version 2014.3. 1996 [17/12/2014]. Available from: <http://www.iucnredlist.org/details/5661/0>.

99. Crocodile Specialist Group. *Alligator sinensis.* The IUCN Red List of Threatened Species. Version 2014.3. 1996. Available from: <http://www.iucnredlist.org/details/867/0>.

100. Revol B. Crocodile farming and conservation, the example of Zimbabwe. Biodivers Conserv. 1995;4(3):299-305.

101. Blake DK, Loveridge JP. The role of commercial crocodile farming in crocodile conservation. Biol Conserv. 1975(September 1974):261-72.

102. Vos Ad. Crocodile conservation in India. Biol Conserv. 1984;29(May 1982):183-9.

103. Group ICS. Farming and the crocodile industry: IUCNCSG; [17/12/2014]. Available from: <http://www.iucncsg.org/pages/farming-and-the-crocodile-industry.html>.

104. Ramo C, Busto B, Utrera A. Breeding and rearing the Orinoco crocodile Crocodylus intermedius in Venezuela. Biol Conserv. 1992;60(2):101-8.

105. Born Free. Fact Sheet: Swimming with dolphins [18/11/2014]. Available from: <http://www.thetravelfoundation.org.uk/images/media/swimming_with_dolphins_01.pdf>.

106. Jason Garcia, Sentinel. O. Attendance slips at SeaWorld parks, but company reports record annual earnings 2014 [18/11/2014]. Available from: <http://articles.orlandosentinel.com/2014-03-13/business/os-seaworld-fourth-quarter-earnings-20140312_1_seaworld-orlando-seaworld-parks-blackfish>.

107. Seaworld. Our position [18/11/2014]. Available from: <http://seaworld.com.au/animals-rides-and-shows/animals/our-position.aspx>.

108. Hammond PS, Bearzi G, Bjørge A, Forney KA, Karkzmarski L, Kasuya T, et al. *Tursiops truncatus*. The IUCN Red List of Threatened Species. Version 2014.3. 2012 [18/11/2014]. Available from: <http://www.iucnredlist.org/details/22563/0>.

109. Born Free. Captive whales and dolphins [18/11/2014]. Available from: <http://www.bornfree.org.uk/campaigns/zoo-check/captive-whales-dolphins/>.

110. CETA Base. Drive fisheries: capture results and information 2014 [18/11/2014]. Available from: <http://www.ceta-base.com/drivefisheries.html>.

111. Vail CS. Socio-economic assessment of marine mammal utilization in the wider Caribbean region: Captivity, viewing and hunting. Regional Workshop of Experts on the Development of the Marine Mammal Action Plan for the Wider Caribbean Region; Bridgetown, Barbados, 18-21 July: United Nations Environment Programme (UNEP); 2005.

112. Curtin S, Wilkes K. Swimming with captive dolphins: current debates and post‐experience dissonance. International Journal of Tourism Research. 2007;9(2):131-46.

113. Constantine R. Increased avoidance of swimmers by wild bottlenose dolphins (*Tursiops truncatus*) due to long‐term exposure to swim‐with‐dolphin tourism. Mar Mamm Sci. 2001;17(4):689-702.

114. Reeves RR, Dawson SM, Jefferson TA, Karczmarski L, Laidre K, O’Corry-Crowe G, et al. *Cephalorhynchus hectori*. The IUCN Red List of Threatened Species. Version 2014.3. 2013 [17/12/2014]. Available from: <http://www.iucnredlist.org/details/4162/0>.

115. Bejder L, Dawson SM, Harraway JA. Responses by Hector's dolphins to boats and swimmers in Porpoise Bay, New Zealand. Mar Mamm Sci. 1999;15(July):738-50.

116. Lundquist D, Gemmell NJ, Würsig B. Behavioural responses of dusky dolphin groups (Lagenorhynchus obscurus) to tour vessels off Kaikoura, New Zealand. PloS one. 2012;7(7):e41969-e.

117. Peters KJ, Parra GJ, Skuza PP, Möller LM. First insights into the effects of swim‐with‐dolphin tourism on the behavior, response, and group structure of southern Australian bottlenose dolphins. Mar Mamm Sci. 2013;29(4):E484-E97.

118. Steckenreuter A, Möller L, Harcourt R. How does Australia's largest dolphin-watching industry affect the behaviour of a small and resident population of Indo-Pacific bottlenose dolphins? J Environ Manag. 2012;97:14-21.

119. Tezanos-Pinto G, Constantine R, Brooks L, Jackson Ja, Mourão F, Wells S, et al. Decline in local abundance of bottlenose dolphins ( Tursiops truncatus ) in the Bay of Islands, New Zealand. Mar Mamm Sci. 2013;29(October):n/a-n/a.

120. Dolphin Research Centre. Our animal family 2014 [17/12/2014]. Available from: <http://www.dolphins.org/our_animal_family?cat=1&id=51>.

121. Dolphin Marine Magic. Our bottlenose dolphins 2014 [17/12/2014]. Available from: <http://www.dolphinmarinemagic.com.au/bottlenose-dolphins>.

122. Dolphin Research Centre. Our history 2014 [17/12/2014]. Available from: <https://dolphins.org/our_history>.

123. Dolphin Marine Magic. Rehabilitation [17/12/2014]. Available from: <http://www.dolphinmarinemagic.com.au/rehabilitation>.

124. Dolphin Research Centre. Conservation 2014 [17/12/2014]. Available from: <http://www.dolphins.org/conservation>.

125. Dolphin Marine Magic. Conservation 2014 [17/12/2014]. Available from: <http://www.dolphinmarinemagic.com.au/conservation>.

126. Dolphin Research Centre. Care facts 2014 [17/12/2014]. Available from: <http://www.dolphins.org/care_facts>.

127. Dublin H, Desai MAA, Hedges MS, Vié J-C, Bambaradeniya C, Lopez MA. Elephant Range States Meeting. 2006.

128. Elephant Action League. Elephants in captivity [19/11/2014]. Available from: <http://elephantleague.org/elephants-in-captivity/>.

129. Choudhury A, Lahiri Choudhury DK, Desai A, Duckworth JW, Easa PS, Johnsingh AJT, et al. (IUCN SSC Asian Elephant Specialist Group) 2008. *Elephas maximus*. The IUCN Red List of Threatened Species. Version 2014.3. Available from: <http://www.iucnredlist.org/details/7140/0>.

130. Blanc J. *Loxodonta africana*. The IUCN Red List of Threatened Species. Version 2014.3. 2008. Available from: <http://www.iucnredlist.org/details/12392/0>.

131. Nijman V. An Assessment of the Live Elephant Trade in Thailand. Cambridge, UK.: 2014.

132. Hayward aD, Mar KU, Lahdenperä M, Lummaa V. Early reproductive investment, senescence and lifetime reproductive success in female Asian elephants. J Evol Biol. 2014;27(4):772-83.

133. Elephant Valley Project. Meet the elephants [15/12/2014]. Available from: <http://www.elephantvalleyproject.org/elephants>.

134. Tong Bai Elephant Tour. Our elephants [15/12/2014]. Available from: <http://tongbai-elephant-tour.com/?page_id=8>.

135. Elephant Nature Park. Elephant Sanctuary Cambodia - Weekly Volunteer [21/11/2014]. Available from: <http://www.elephantnaturepark.org/enp/en/22-elephant-sanctuary-cambodia-weekly-volunteer>.

136. Gibbon Spotting Cambodia. Code of conduct [15/12/2014]. Available from: <http://www.gibbonspottingcambodia.com/app/code-of-conduct/>.

137. Brockelman W, Geissmann T, Timmins T, Traeholt C. *Hylobates pileatus.* The IUCN Red List of Threatened Species. Version 2014.3. 2008 [15/12/2014]. Available from: <http://www.iucnredlist.org/details/10552/0>.

138. Gibbon Spotting Cambodia. Gibbon Spotting Cambodia partnerships [15/12/2014]. Available from: <http://www.gibbonspottingcambodia.com/app/gibbon-spotting-cambodia-partnerships/>.

139. Expert Africa. Gorilla trekking safaris in Rwanda [15/12/2014]. Available from: <http://www.expertafrica.com/rwanda/info/gorilla-trekking-safaris-in-rwanda>.

140. Robbins M, Williamson L. *Gorilla beringei*. The IUCN Red List of Threatened Species. Version 2014.3. 2008 [15/12/2014]. Available from: <http://www.iucnredlist.org/details/39994/0>.

141. Walsh PD, Tutin CEG, Oates JF, Baillie JEM, Maisels F, Stokes EJ, et al. *Gorilla gorilla.* The IUCN Red List of Threatened Species. Version 2014.3. 2008 [15/12/2014]. Available from: <http://www.iucnredlist.org/details/9404/0>.

142. On The Go Tours. Mountain gorilla trekking in Africa [15/12/2014]. Available from: <http://www.onthegotours.com/Africa/gorilla-trekking>.

143. Adams WM, Infield M. Who is on the gorilla’s payroll? Claims on tourist revenue from a Ugandan National Park. World Development. 2003;31(1):177-90.

144. Sandbrook CG. Local economic impact of different forms of nature-based tourism. Conservation Letters. 2010;3(1):21-8.

145. The Corner of Here and Now. A baby gorilla touched my leg 2014. Available from: <http://cornerofhereandnow.wordpress.com/2014/09/09/a-baby-gorilla-touched-my-leg/>.

146. Homsy J. Ape tourism and human diseases: how close should we get. International Gorilla Conservation. 1999(February).

147. Wallis J, Lee DR. Primate conservation: the prevention of disease transmission. International Journal of Primatology. 1999;20(6):803-26.

148. AGB Films. The Hyena Men [17/12/2014]. Available from: <http://www.agbfilms.co.uk/Current/hyena-men.html>.

149. CCTV.com. Faces of Africa 04/29/2013 The hyena men 2013 [17/12/2014]. Available from: <http://english.cntv.cn/program/facesofafrica/20130429/100402.shtml>.

150. IUCN1 H. [17/12/2014]. Available from: <http://www.iucnredlist.org/details/5674/0>.

151. Borchert P. Is walking with lions good conservation? Probably not. 2013 [21/11/2014]. Available from: <http://africageographic.com/blog/walking-with-lions-good-conservation-probably-not/>.

152. Bauer H, Nowell K, Packer C. *Panthera leo*. The IUCN Red List of Threatened Species. Version 2014.3. 2012 [20/11/2014]. Available from: <http://www.iucnredlist.org/details/15951/0>.

153. Alert. African lion rehabilitation and release into the wild program [20/11/2014]. Available from: <http://www.lionalert.org/alert/project-detail/african-lion-rehabilitation--release-into-the-wild-program>.

154. Hunter LTB, White P, Henschel P, Frank L, Burton C, Loveridge A, et al. Walking with lions: why there is no role for captive-origin lions Panthera leo in species restoration. Oryx. 2012;47(01):19-24.

155. Hunter LTB. Walking with Lions: The Myth of Conservation 2014 [21/11/2014]. Available from: <http://www.panthera.org/blog/walking-lions-myth-conservation>.

156. Lions Tigers and Bears. [17/12/2014]. Available from: <http://lionstigersandbears.org/about-us/educational-group-visits>.

157. Big Cat Rescue. Day Tours [17/12/2014]. Available from: <http://bigcatrescue.org/day-tours/>.

158. The Wild Animal Sanctuary. Get to know our animals [17/12/2014]. Available from: <http://www.wildanimalsanctuary.org/animalstories/africanlions.html#!animals/criz>.

159. Lion Rescue. [17/12/2014]. Available from: <http://www.lionrescue.org.za/ourlions.htm#top>.

160. The Wildcat Sanctuary. [17/12/2014]. Available from: <http://www.wildcatsanctuary.org/residents/big-cats/lions/>.

161. Big Cat Rescue. [17/12/2014]. Available from: <http://bigcatrescue.org/visit-the-big-cats/>.

162. Big Cat Rescue. Breeding and selling [17/12/2014]. Available from: <http://bigcatrescue.org/breeding-and-selling/>.

163. Baskin C. Our evolution [17/12/2014]. Available from: <http://bigcatrescue.org/about/our-evolution/>.

164. YOSL-OIC. Chapter 6 - Sumatran Orangutan Viewing Guidelines. 2009 [cited 18/12/2014]. In: Guidebook to the Gunung Leuser National Park [Internet]. Orangutan Information Centre. Medan. Indonesia., [cited 18/12/2014]. Available from: <http://www.orangutans-sos.org/resources/guidebook>.

165. Singleton I, Wich SA, Griffiths M. *Pongo abelii*. The IUCN Red List of Threatened Species. Version 2014.3. 2008 [14/11/2014]. Available from: <http://www.iucnredlist.org/details/39780/0>.

166. Ancrenaz M, Marshall A, Goossens B, van Schaik C, Sugardjito J, Gumal M, et al. *Pongo pygmaeus*. The IUCN Red List of Threatened Species. Version 2014.3. 2008 [17/12/2014]. Available from: <http://www.iucnredlist.org/details/17975/0>.

167. Orangutan Appeal UK. Meet the orangutans [17/12/2014]. Available from: <http://www.orangutan-appeal.org.uk/about-us/meet-the-orangutans>.

168. Sumatran Orangutan Society. Our projects [17/12/2014]. Available from: <http://www.orangutans-sos.org/projects>.

169. The Borneo Orangutan Survival Foundation. Developing Batikap 2014 [21/01/2015]. Available from: <http://orangutan.or.id/developing-batikap/>.

170. Sumatran Orangutan Society. Environmental Education [17/12/2014]. Available from: <http://www.orangutans-sos.org/projects/education>.

171. Orangutan Appeal UK. Education projects for schools and families [17/12/2014]. Available from: <http://www.orangutan-appeal.org.uk/about-us/education-program>.

172. Churchill E. FAQ [17/12/2014]. Available from: <http://www.everythingchurchill.com/plan-your-adventure/frequently-asked-questions-faqs/#faq6>.

173. Stewart EJ, Draper D, Johnston ME. A Review of Tourism Research in the Polar Regions. 2005;58(4):383-94.

174. Dyck MG, Baydack RK. Human Activities Associated with Polar Bear Viewing Near Churchill, Manitoba, Canada. Human Dimensions of Wildlife. 2006;11(2):143-5.

175. Schliebe S, Wiig Ø, Derocher A, Lunn N. (IUCN SSC Polar Bear Specialist Group) *Ursus maritimus*. The IUCN Red List of Threatened Species. Version 2014.3. 2008 [17/12/2014]. Available from: <http://www.iucnredlist.org/details/22823/0>.

176. Lemelin RH, Wiersma EC. Perceptions of polar bear tourists: A qualitative analysis. Human Dimensions of Wildlife. 2007;12(1):45-52.

177. Isaacs JC. The limited potential of ecotourism to contribute to wildlife conservation. Wildl Soc Bull. 2000;28(1):61-9.

178. Cayman Turtle Farm. FAQ [17/12/2014]. Available from: <http://www.turtle.ky/faq>.

179. Broadus J. Ecotourism on Isla Mujeres: Save the Turtles [17/12/2014]. Available from: <http://vagabond3.com/ecotourism-on-isla-mujeres-save-the-turtles/>.

180. Cayman Turtle Farm. History [17/12/2014]. Available from: <http://www.turtle.ky/history-history>.

181. Seminoff JA. (Southwest Fisheries Science Center, U.S.) *Chelonia mydas*. The IUCN Red List of Threatened Species. Version 2014.3. 2004 [17/12/2014]. Available from: <http://www.iucnredlist.org/details/4615/0>.

182. Bell CDL, Parsons J, Austin TJ, Broderick AC, Ebanks-Petrie G, Godley BJ. Some of them came home: the Cayman Turtle Farm headstarting project for the green turtle Chelonia mydas. Oryx. 2005;39(02):137-48.

183. D’Cruze N, Alcock R, Donnelly M. The Cayman Turtle Farm: why we can’t have our green turtle and eat it too. J Agric Environ Ethics. 2014.

184. Warwick C, Arena PC, Steedman C. Health implications associated with exposure to farmed and wild sea turtles. JRSM short reports. 2013;4(1):8-.

185. Clua E, Buray N, Legendre P, Mourier J, Planes S. Behavioural response of sicklefin lemon sharks Negaprion acutidens to underwater feeding for ecotourism purposes. Mar Ecol Prog Ser. 2010;414:257-66.

186. Evans A. Why I Won’t Go Shark Cage Diving 2013 [17/12/2014]. Available from: <http://digitalnomad.nationalgeographic.com/2013/11/21/why-i-wont-go-shark-cage-diving/>.

187. Fergusson I, Compagno LJV, Marks M. *Carcharodon carcharias*. The IUCN Red List of Threatened Species. Version 2014.3 2009 [17/12/2014]. Available from: <http://www.iucnredlist.org/details/3855/0>.

188. Sundström LF. *Negaprion brevirostris*. The IUCN Red List of Threatened Species. Version 2014.3. 2009 [17/12/2014]. Available from: <http://www.iucnredlist.org/details/39380/0>.

189. Hammerschlag N, Gallagher AJ, Wester J, Luo J, Ault JS. Don’t bite the hand that feeds: assessing ecological impacts of provisioning ecotourism on an apex marine predator. Funct Ecol. 2012;26(3):567-76.

190. Laroche RK, Kock AA, Dill LM, Oosthuizen WH. Effects of provisioning ecotourism activity on the behaviour of white sharks *Carcharodon carcharias*. Marine Ecology Progress …. 2007;338(Orams 2002):199-209.

191. Lawson A. Snake charmers fight for survival 2003 [17/12/2014]. Available from: <http://news.bbc.co.uk/1/hi/world/south_asia/2733039.stm>.

192. Stuart B, Wogan G, Grismer L, Auliya M, Inger RF, Lilley R, et al. *Ophiophagus hannah*. The IUCN Red List of Threatened Species. Version 2014.3. 2012 [17/12/2014]. Available from: <http://www.iucnredlist.org/details/177540/0>.

193. Srinivasulu C, Srinivasulu B, Deepak V, Achyuthan NS, Das A, Kulkarni NU. *Trimeresurus gramineus*. The IUCN Red List of Threatened Species. Version 2014.3. 2013 [17/12/2014]. Available from: <http://www.iucnredlist.org/details/178245/0>.

194. Stuart B, Nguyen TQ, Thy N, Grismer L, Chan-Ard T, Iskandar D, et al. *Python bivittatus*. The IUCN Red List of Threatened Species. Version 2014.3. 2012 [17/12/2014]. Available from: <http://www.iucnredlist.org/details/193451/0>.

195. Wikipaedia. Snake charming [17/12/2014]. Available from: <http://en.wikipedia.org/wiki/Snake_charming>.

196. Jakarta Animal Aid Network. Aid for dancing monkeys. Available from: <http://jakartaanimalaid.com/blog/?page_id=5800>.

197. Rayda N. City’s former dancing monkeys now seeking their own isle of refuge: The Jakarta Globe; 2013 [14/11/2014]. Available from: <http://thejakartaglobe.beritasatu.com/features/citys-former-dancing-monkeys-now-seeking-their-own-isle-of-refuge/>.

198. Big Cat Rescue. China raises tigers like battery hens [17/12/2014]. Available from: <http://bigcatrescue.org/china-raises-tigers-like-battery-hens/>.

199. Chundawat RS, Habib B, Karanth U, Kawanishi K, Ahmad Khan J, Lynam T, et al. *Panthera tigris*. The IUCN Red List of Threatened Species. Version 2014.3 2011 [19/11/2014]. Available from: <http://www.iucnredlist.org/details/15955/0>.

200. Abbott B, van Kooten GC. Can domestication of wildlife lead to conservation? The economics of tiger farming in China. Ecol Econ. 2011;70(4):721-8.

201. Kirkpatrick RC, Emerton L. Killing tigers to save them: fallacies of the farming argument. Conservation biology : the journal of the Society for Conservation Biology. 2010;24(3):655-9.

202. Huahin H. Tiger Temple and River Kwai [19/11/2014]. Available from: <http://holidayshuahin.com/tour/tiger-temple-river-kwai/>.

203. Phuket TK. [19/11/2014]. Available from: <http://www.tigerkingdom.com/phuket-3/>.

204. Care For The Wild International. Exploiting the tiger: Illegal trade, animal cruelty and tourists at risk at the Tiger Temple 2008 [19/11/2014]. Available from: <http://www.careforthewild.com/wp-content/uploads/2012/05/tigertemplereport08_final_v11.pdf>.

205. Loeffler I, Robinson J, Cochrane G. Compromised health and welfare of bears farmed for bile in China. Anim Welf. 2009;18(3):225-35.

206. Nogami F. Japan's Bear Parks: ALIVE; 2001 [14/11/2014]. Available from: <http://www.alive-net.net/english/en-zoocheck/bearpark/bear-noga1.html>.

207. Lynn G, Rogers C. Civet cat coffee's animal cruelty secrets: BBC News, London; 2013 [18/11/2014]. Available from: <http://www.bbc.co.uk/news/uk-england-london-24034029>.

208. PETA Asia Pacific. Civets suffer for vile Kopi Luwak Coffee [18/11/2014]. Available from: <http://action.petaasiapacific.com/ea-action/action?ea.client.id=110&ea.campaign.id=22769>.

209. Bolton M. Captive Breeding and the management of adult crocodiles. FAO Conservation Guide 22: The management of crocodiles in captivity: Food and Agriculture Organization of the United Nations; 1989.

210. Waples KA, Gales NJ. Evaluating and minimising social stress in the care of captive bottlenose dolphins (*Tursiops aduncus*). Zoo Biol. 2002;21(1):5-26.

211. Hughes P. Animals, values and tourism—structural shifts in UK dolphin tourism provision. Tourism Management. 2001;22(4):321-9.

212. Lundquist D, Gemmell N, Würsig B, Markowitz T. Dusky dolphin movement patterns: short-term effects of tourism. N Z J Mar Freshw Res. 2013;47(4):430-49.

213. Dolphin Research Centre. Volunteer at Dolphin Research Centre [17/12/2014]. Available from: <https://dolphins.org/volunteer?aid=8>.

214. Dolphin Marine Magic. Caring for our animals [17/12/2014]. Available from: <http://www.dolphinmarinemagic.com.au/caring>.

215. Brensing K. Approaches to the behavior of dolphins *Tursiops truncatus* during unstructured swim-with-dolphin programs. Unveröffentlichte Dissertation, Freie Universität Berlin. 2004.

216. Brensing K, Linke K, Busch M, Matthes I, van der Woude SE. Impact of different groups of swimmers on dolphins in swim-with-the-dolphin programs in two settings. Anthrozoos: A Multidisciplinary Journal of The Interactions of People & Animals. 2005;18(4):409-29.

217. Kontogeorgopoulos N. Wildlife tourism in semi-captive settings: a case study of elephant camps in northern Thailand. Current Issues in Tourism. 2009;12:429-49.

218. Hile J. Activists Denounce Thailand's Elephant "Crushing" Ritual: National Geographic News; 2002 [19/11/2014]. Available from: <http://news.nationalgeographic.com/news/2002/10/1016_021016_phajaan.html>.

219. Elephant Valley Project. FAQ [15/12/2014]. Available from: <http://www.elephantvalleyproject.org/about/faq/>.

220. Shannon G, Page B, Slotow R, Duffy K. African elephant home range and habitat selection in Pongola Game Reserve, South Africa. Afr Zool. 2006;41(April):37-44.

221. Journeys International. Gorilla trekking FAQ [15/12/2014]. Available from: <http://www.journeysinternational.com/gorilla-trekking-faq#7>.

222. Campaign Against Canned Hunting. The life of a captive bred lion in South Africa [21/11/2014]. Available from: <http://www.cannedlion.org/cub-petting.html>.

223. Barkham P. 'Canned hunting': the lions bred for slaughter The Guardian; 2013 [21/11/2014]. Available from: <http://www.theguardian.com/environment/2013/jun/03/canned-hunting-lions-bred-slaughter>.

224. Lionsrock Big Cat Sanctuary. Feeding [17/12/2014]. Available from: <http://www.lionsrock.org/lionsrock/care/feeding/>.

225. Lionsrock Big Cat Sanctuary. Special care unit at Lionsrock [17/12/2014]. Available from: <http://www.lionsrock.org/lionsrock/care/special-care-unit-at-lionsrock-/>.

226. Lionsrock Big Cat Sanctuary. Medical checks and enrichment [17/12/2014]. Available from: <http://www.lionsrock.org/lionsrock/care/medical-checks-and-enrichment/>.

227. Samboja Lodge BOS. BOS Samboja Lestari project [22/01/2015]. Available from: <http://www.sambojalodge.com/AboutBOSFoundation/BOSSambojaLestari/>.

228. Karplus I. Social control of growth in *Macrobrachium rosenbergii* (De Man): a review and prospects for future research. Aquac Res. 2005;36:238-54.

229. Dyck MG, Baydack RK. Vigilance behaviour of polar bears (*Ursus maritimus*) in the context of wildlife-viewing activities at Churchill, Manitoba, Canada. Biol Conserv. 2004;116(3):343-50.

230. Mohanty G. Adivasi. Journal of he scheduled castes and scheduled tribes. 2004;44(1):1-104.

231. Flintoff C. In India, snake charmers are losing their sway: NPR.org; 2011 [17/12/2014]. Available from: <http://www.npr.org/2011/08/08/139086119/in-india-snake-charmers-are-losing-their-sway>.

232. Jones R. Exposed: Dark secret of the farm where tigers' bodies are plundered to make £185 wine: The Daily Mail; 2010 [17/12/2014]. Available from: <http://www.dailymail.co.uk/news/article-1252500/Exposed-Dark-secret-farm-tigers-bodies-plundered-make-185-wine.html>.
