# Supplementary material for: The Customer Isn't Always Right—Conservation and Animal Welfare Implications of the Increasing Demand for Wildlife Tourism
Source: PLoS One. 2015 Oct 21;10(10):e0138939. doi: 10.1371/journal.pone.0138939 (PMC4619427; doi:10.1371/journal.pone.0138939)
Supplement: S1 Table — (DOCX) [file pone.0138939.s004.docx]

**S1 Table.**

| **WTA category** | **Wild or captive?** | **Attraction's primary (actual) focus?** | **Primary source of revenue** | **Permanent venue?** | **Typically single or multiple taxon?** |
| --- | --- | --- | --- | --- | --- |
| Wild attractions | Wild | Tourist revenue | Tourism | Varies | Single |
| Sanctuary attractions | Captive (often free to roam) | Improved welfare of subject individuals | Tourism | Yes | Varies |
| Farmed wildlife attractions | Captive | Tourist revenue | Semi-consumptive provision of animal products | Yes | Single |
| Captive interactions | Captive | Tourist revenue | Tourism | Yes | Single |
| Street performance | Captive | Tourist revenue | Tourism | No | Single |
| Special cases | Captive | Tourist revenue | Tourism | Varies | Multiple |
