# Supplementary material for: The Customer Isn't Always Right—Conservation and Animal Welfare Implications of the Increasing Demand for Wildlife Tourism
Source: PLoS One. 2015 Oct 21;10(10):e0138939. doi: 10.1371/journal.pone.0138939 (PMC4619427; doi:10.1371/journal.pone.0138939)
Supplement: S2 Table — See S2 Appendix for reference citations. (DOCX) [file pone.0138939.s005.docx]

**S2 Table.**

|  | **Wildlife Tourist Attraction type** | **Category** | **Consumptive or non-consumptive** | **Description of tourist experience** | **Example reference** |
| --- | --- | --- | --- | --- | --- |
| 1 | Animals in circuses | Special case | Non-consumptive | Public performances, show (acrobatics by animals and using animals). | [7] |
| 2 | Bear baiting | Special case | Consumptive | Public performances, show (dogs attack a chained bear until it dies). | [8] |
| 3 | Bear bile farm | Farmed wildlife attraction | Semi-consumptive | Observation, bile extraction, bile for sale. | [9] |
| 4 | Bear dancing | Street performance | Non-consumptive | Observation, public performance (bears move due to aversive conditioning with the tugs of a rope through their nose and mouth to encourage by passers to donate money). | [10] |
| 5 | Bear parks | Captive interaction | Non-consumptive | Observe, feed, public performance (football, basketball, maths), museum. | [11] |
| 6 | Bear sanctuary | Sanctuary attraction | Non-consumptive | School education programme, observation. | [12, 13] |
| 7 | Bird watching tours | Wild attraction | Non-consumptive | Observe multiple species of bird in the wild | [14, 15] |
| 8 | Civet coffee farm | Farmed wildlife attraction | Semi-consumptive | Observation, coffee tasting, tour around the coffee plantation. | [16] |
| 9 | Coral reef diving | Wild attraction | Non-consumptive | Observe coral reefs and associated biota. | [17] |
| 10 | Crocodile farm | Farmed wildlife attraction | Semi-consumptive | Observation, public performance (wrestling show), feeding, crocodile products for sale. | [18] |
| 11 | Dolphin interactions (captive) | Captive interaction | Non-consumptive | Public interaction (swim, play, free time), public performance. | [19, 20] |
| 12 | Dolphin interactions (wild) | Wild attraction | Non-consumptive | Observation, dive and swim alongside dolphins in the wild. | [21, 22] |
| 13 | Dolphin sanctuary | Sanctuary attraction | Non-consumptive | Observation, Interaction (swim, play), feeding, education. | [23] |
| 14 | Dolphin watching | Wild attraction | Non-consumptive | Observe dolphins in the wild. | [24] |
| 15 | Elephant parks | Captive interaction | Non-consumptive | Observation, public interaction (rides, walks, bathing), tour, feeding, public performance. | [25-27] |
| 16 | Elephant sanctuary | Sanctuary attraction | Non-consumptive | Observation, walking, feeding, public interaction (bathing). | [28, 29] |
| 17 | Fur seal watching | Wild attraction | Non-consumptive | Observe fur seals in the wild. | [30] |
| 18 | Gannet watching | Wild attraction | Non-consumptive | Observe gannets in wild colonies | [31] |
| 19 | Gibbon watching | Wild attraction | Non-consumptive | Observe habituated gibbons in the wild. | [32] |
| 20 | Gorilla watching | Wild attraction | Non-consumptive | Observe habituated gorillas in the wild. | [33, 34] |
| 21 | Hyena men in Nigeria | Street performance | Non-consumptive | Observation, public performance (in villages to encourage purchase of traditional medicine). | [35, 36] |
| 22 | Kiwi watching tours | Wild attraction | Non-consumptive | Observe kiwis in the wild. | [37] |
| 23 | Lion interaction | Captive interaction | Non-consumptive | Observation, walking and interacting (holding cubs, touching and observing in the lion enclosures). | [38-40] |
| 24 | Lion sanctuary | Sanctuary attraction | Non-consumptive | Observing, feeding, education. | [41, 42] |
| 25 | Little penguin watching | Wild attraction | Non-consumptive | Observe little penguins in the wild | [43] |
| 26 | Macaque shows, training and ‘Monkey schools’ (Thailand) | Captive attraction | Non-consumptive | Observation, public performance (dancing, display while costumed), ‘training’. | [44] |
| 27 | Meerkat watching | Wild attraction | Non-consumptive | Observe meerkats in the wild. | [45] |
| 28 | Monarch butterfly watching | Wild attraction | Non-consumptive | Observe the annual migration of Monarch butterflies in Mexico | [46] |
| 29 | Orang-utan sanctuary | Sanctuary attraction | Non-consumptive | Observing orang-utans in enclosures. | [47] |
| 30 | Photo props | Special case | Non-consumptive | Observation, public interaction (animals conditioned to enable photos with tourists). | [48] |
| 31 | Pink river dolphin watching | Wild attraction | Non-consumptive | Observe pink river dolphins in the wild. | [49, 50] |
| 32 | Polar bear watching | Wild attraction | Non-consumptive | Observe polar bears in the wild. | [51, 52] |
| 33 | Rattle snake roundup | Special case | Consumptive | Pursuit and slaughter, skinning competition, eating competition, purchase of items made out of snake parts. | [53, 54] |
| 34 | Reindeer prop | Captive interaction | Non-consumptive | (Reindeer are used for as props for Christmas markets and fairs.) Observation, public interaction. | [55] |
| 35 | Rhino sanctuary | Sanctuary attraction | Non-consumptive | Observe rhinos in enclosures, education. | [56] |
| 36 | River otter watching | Wild attraction | Non-consumptive | Observe giant river otters in the wild. | [57] |
| 37 | Royal albatross watching | Wild attraction | Non-consumptive | Observe royal albatross at a wild colony | [58] |
| 38 | Safari | Wild attraction | Non-consumptive | Observe many different taxa in the wild. | [59, 60] |
| 39 | Sea turtle farm | Farmed wildlife attraction | Semi-consumptive | Observation, public interaction (touch, handling, swimming with). | [61] |
| 40 | Shark cage diving | Wild attraction | Non-consumptive | Observe sharks in the wild, feed / bait to attract sharks. | [62, 63] |
| 41 | Slow loris sanctuary | Sanctuary attraction | Non-consumptive | Observation, guided tour and educational presentation. | [64] |
| 42 | Snake charming | Street performance | Non-consumptive | Observation, public performance (to encourage by passers to donate money). | [65] |
| 43 | Snake farms | Farmed wildlife attraction | Semi-consumptive | Public performances, venom collection. | [66, 67] |
| 44 | Street dancing macaques (Indonesia) | Street performance attraction | Non-consumptive | Observation, public performance, (costumed - performing macaques encourage tourists to buy from street vendors). | [68] |
| 45 | Tiger farm | Farmed wildlife attraction | Semi-consumptive | Tiger bone wine, skins and other products for sale, observation, public performances, (hunting live animals in enclosure). | [69] |
| 46 | Tiger interactions | Captive interaction | Non-consumptive | Public interaction (play and exercise with cubs and adults), observation, feeding, washing, photo opportunities. | [70] |
| 47 | Tiger sanctuary | Sanctuary attraction | Non-consumptive | Observation, guided tours, education. | [71, 72] |
| 48 | Whale watching | Wild attraction | Non-consumptive | Observe whales in the wild. | [73] |
