# Supplementary material for: The Customer Isn't Always Right—Conservation and Animal Welfare Implications of the Increasing Demand for Wildlife Tourism
Source: PLoS One. 2015 Oct 21;10(10):e0138939. doi: 10.1371/journal.pone.0138939 (PMC4619427; doi:10.1371/journal.pone.0138939)
Supplement: S3 Table — WTA types are listed alphabetically. See S2 Appendix for reference citations. (DOCX) [file pone.0138939.s006.docx]

**S3 Table**

|  | **Bear bile farms** | | | | |
| --- | --- | --- | --- | --- | --- |
|  | | Criterion | Score | Justification / details | Supporting References |
|  |  | Animals wild or captive? | Captive | - | [74] |
| Accessibility | | No. animals in attraction | 12,500 at lowest but estimates of up to 20-40,000 | Estimate. | [75] |
|  |  | No. of tourists | Inestimable, but probably substantial. | In 2008 1,500 tourists visited a single Korean bear bile farm in 10 days. A Chinese bear breeding (for bear bile farms) centre receives 300,000 visitors p/a. | [76, 77] |
| Conservation | | IUCN population status | VU | Most common species is Asiatic black bear (*Ursus thibetanus*). | [74]  [78] |
|  |  | Animals sourced from the wild? | Yes | Bears are still captured from the wild, despite the existence of farms for the breeding of captive bears. | [78, 79] |
|  |  | Evidence of direct conservation benefit | No | Claims that farms diminish impacts on wild populations are unlikely to be accurate. | [78, 79] |
|  |  | Evidence of indirect benefit | No | None | - |
| Welfare | | Intention to promote welfare? | No | - | - |

A

B

|  | 1. **Bear dancing** | | | |  |
| --- | --- | --- | --- | --- | --- |
|  | 1. Criterion | 1. Score | 1. Justification / details | 1. Supporting Reference | |
|  | 1. Animals wild or captive? | 1. Captive | 1. - | 1. - | |
| 1. Accessibility | 1. No. animals in attraction | 1. Unknown | 1. Attraction occurs in India, Pakistan, Sri Lanka and Bangladesh. 1200 dancing bears estimated in India in 2002, evidence of at least 28 individuals in 2010. Recent claims suggest that bear dancing in India has ceased. | 1. [10, 80, 81] | |
|  | 1. No. of tourists | 1. Unknown, but presumed highly accessible. | 1. Geographical spread (see above) may suggest a high encounter rate with tourists. |  | |
| 1. Conservation | 1. IUCN population status | 1. LC / VU | 1. Brown bears (*Ursus arctos; LC*), Asiatic black bears (*Ursus thibetanus; VU*) and sloth bears (*Melursus ursinus; VU*) known to be used. | 1. [78, 82, 83] [84] | |
|  | 1. Animals sourced from the wild? | 1. Yes | 1. Cubs typically poached from wild | 1. [82, 84] | |
|  | 1. Evidence of direct conservation benefit | 1. No | 1. - | 1. - | |
|  | 1. Evidence of indirect benefit | 1. No | 1. No educational benefit. | 1. - | |
| 1. Welfare | 1. Intention to promote welfare? | 1. No | 1. - | 1. - | |

C

|  | **Bear parks** | | | |  |
| --- | --- | --- | --- | --- | --- |
|  | Criterion | Score | Justification / details | Supporting References | |
|  | Animals wild or captive? | Captive | - | - | |
| Accessibility | No. animals in attraction | Up to 400 | 200 known to be in one park. | [85] | |
|  | No. of tourists | Unknown but likely to be substantial | Figures for attendance not available, but, for example, the city of Noboribetsu receives 3 million visitors p/a, and the bear park is a seven minute trip from the city. | [86] | |
| Conservation | IUCN population status | LC-VU | Both brown bears (*Ursus arctos*) and Asiatic black bears (*[Ursus thibetanus](http://www.iucnredlist.org/details/22824/0)*) are kept in Japanese bear parks | [78, 83] | |
|  | Animals sourced from the wild? | No | Evidence scant. Bears were initially introduced as orphaned cubs, but are now bred in captivity. | [87] | |
|  | Evidence of direct conservation benefit | No | - | - | |
|  | Evidence of indirect benefit | No | No conservation educational initiatives recorded. | - | |
| Welfare | Intention to promote welfare? | No | Original intention was to provide for orphaned cubs, but continued breeding and poor conditions suggest principal motive is tourist revenue | [85] | |

|  | Bear sanctuaries | | | |  |
| --- | --- | --- | --- | --- | --- |
|  | Criterion | Score | Justification / details | Supporting References | |
|  | Animals wild or captive? | Captive | - | - | |
| Accessibility | No. animals in attraction | 3,000-5,000 | One organisation alone has taken in 1,744 bears. | [88] | |
|  | No. of tourists | >10 million | There are at least 18 bear sanctuaries worldwide with some receiving more than 800,000 visitors annually. | [89] | |
| Conservation | IUCN population status | LC-VU | Brown bear (*[Ursus arctos](http://www.iucnredlist.org/details/41688/0)*) (LC), Asiatic black bear (*[Ursus thibetanus](http://www.iucnredlist.org/details/22824/0)*) (VU), Sloth bear (*[Melursus ursinus](http://www.iucnredlist.org/details/13143/0)*) (VU). | [78, 82, 83] [84] | |
|  | Animals sourced from the wild? | No | Animals are sourced from other captive attractions. | - | |
|  | Evidence of direct conservation benefit | No | Bears are unlikely to be reintroduced due to their captive past. | [89] | |
|  | Evidence of indirect benefit | Yes | Sanctuaries are often part of conservation schemes promoting conservation through education. | [89] | |
| Welfare | Intention to promote / increase welfare? | Yes | The aim of sanctuaries is to provide better welfare for animals that have been kept in captivity. | [89] | |

D

**E**

|  | 1. **Civet coffee farms** | | | |  |
| --- | --- | --- | --- | --- | --- |
|  | 1. Criterion | 1. Score | 1. Justification / details | 1. Supporting References | |
|  | 1. Animals wild or captive? | 1. Captive | 1. - | 1. - | |
| 1. Accessibility | 1. No. animals in attraction | 1. 1700-10,000 | 1. Thousands of individual civets estimated to be poached from the wild for coffee industry p/a. One article claims that 240 civets are used on one farm to produce 7 tonnes of coffee p/a, and another 120 to produce 500 kg. World production approximates 50 tonnes, suggesting at least 1,700 civets globally. | 1. [16, 90, 91] | |
|  | 1. No. of tourists | 1. Unknown |  |  | |
| 1. Conservation | 1. IUCN population status | 1. LC/VU | 1. Most commonly common palm civets (*[Paradoxurus hermaphroditus](http://www.iucnredlist.org/details/41693/0)*) (LC) but also binturong (*Arctictis binturong*) (VU). | 1. [90] {Duckworth, 2008 #91, 92] | |
|  | 1. Animals sourced from the wild? | 1. Yes | 1. A large proportion of civets used are thought to be poached from the wild. | 1. [90] | |
|  | 1. Evidence of direct conservation benefit | 1. No | 1. - | 1. - | |
|  | 1. Evidence of indirect benefit | 1. No | 1. No educational benefit. | 1. - | |
| 1. Welfare | 1. Intention to promote welfare? | 1. No | 1. - | 1. - | |

F

|  | 1. **Crocodile farms** | | | |  |
| --- | --- | --- | --- | --- | --- |
|  | 1. Criterion | 1. Score | 1. Justification / details | 1. Supporting References | |
|  | 1. Animals wild or captive? | 1. Captive | 1. - | 1. - | |
| 1. Accessibility | 1. No. animals in attraction | 1. 1,000,000 | 1. 500 farms in 47 countries globally with approximately 1 million crocodiles and alligators. | 1. [93] | |
|  | 1. No. of tourists | 1. >2,500,000 | 1. One alligator farm in Florida is thought to attract 200,000 visitors a year. | 1. [94] | |
| 1. Conservation | 1. IUCN population status | 1. LC-CE | 1. American alligator ([*Alligator mississippiensis*](http://www.iucnredlist.org/details/46583/0)) (LC); American crocodile (*Crocodilurus amazonicus*) (LC); Indian Gavial (*Gavialis gangeticus*) (CE); Nile crocodile (*Crocodylus niloticus*) (LC); Orinoco crocodile (*Crocodylus intermedius*) (CE); Chinese alligator (*Alligator* *sinensis*) (CE); Siamese crocodile (*Crocodylus siamensis*) (CE). | 1. [95-99] | |
|  | 1. Animals sourced from the wild? | 1. Yes, partly. | 1. It is illegal to take eggs from the wild from species that are at risk. It is uncertain whether illegal collection occurs, but collecting eggs from species with large populations is allowed and does occur. | 1. [93] [100] [101] [102] | |
|  | 1. Evidence of direct conservation benefit | 1. No | 1. - | 1. - | |
|  | 1. Evidence of indirect benefit | 1. Yes | 1. There is evidence that crocodile farming may reduce the pressure on wild crocodile populations. | 1. [103, 104] 2. [101] | |
| 1. Welfare | 1. Intention to promote / increase welfare? | 1. No | 1. As the crocodiles are farmed for their skin and meat the intention is not to increase the welfare for the animals. | 1. - | |

G

|  | 1. **Dolphin interactions (captive)** | | | |  |
| --- | --- | --- | --- | --- | --- |
|  | 1. Criterion | 1. Score | 1. Justification / details | 1. Supporting Reference | |
|  | 1. Animals wild or captive? | 1. Captive | 1. - | 1. - | |
| 1. Accessibility | 1. No. animals in attraction | 1. >1000 | 1. In 2005, estimates for American and the Caribbean were 730 dolphins in captivity (these comprising 71% of all attractions). | 1. [105] | |
|  | 1. No. of tourists | 1. >24 million | 1. USA company Seaworld (six parks) alone hosted >24 million visitors in 2013. | 1. [106] | |
| 1. Conservation | 1. IUCN population status | 1. LC | 1. Bottlenosed dolphin (*Tursiops truncatus*) most usual species. | 1. [107] 2. [108] | |
|  | 1. Animals sourced from the wild? | 1. Yes | 1. Captive breeding programs supply dolphins, but wild dolphins are still captured and sold for captive swim attractions. | 1. [109, 110] | |
|  | 1. Evidence of direct conservation benefit | 1. No | 1. - | 1. - | |
|  | 1. Evidence of indirect benefit | 1. Little | 1. These attractions offer education and promote research, but may distort public perception of the marine environment; captive studies may be irrelevant to dolphin conservation. | 1. [111, 112] | |
| 1. Welfare | 1. Intention to promote / increase welfare? | 1. No | 1. - | 1. - | |

H

|  | 1. **Dolphin interactions (wild)** | | | |  |
| --- | --- | --- | --- | --- | --- |
|  | 1. Criterion | 1. Score | 1. Justification / details | 1. Supporting Reference | |
|  | 1. Animals wild or captive? | 1. Wild | 1. The tourist attractions take place in the wild. | 1. - | |
| 1. Accessibility | 1. No. animals in attraction | 1. Up to 126,000 | 1. Attractions are common around the coast of America, Belize and New Zealand where there are approximately 126,000 common bottlenose dolphins. | 1. [108] | |
|  | 1. No. of tourists | 1. 32,000 | 1. 42 boats (ca 12 visitors each) per week have been recorded visiting dolphins along the coast of New Zealand. There are approximately 15 dolphin interaction operators in New Zealand and Australia. | 1. [113] | |
| 1. Conservation | 1. IUCN population status | 1. LC-EN | 1. Common bottlenose dolphin (*Tursiops truncatus*) (LC); Hector's dolphin (*Cephalorhynchus hectori*) (EN) | 1. [108, 114] | |
|  | 1. Animals sourced from the wild? | 1. No | 1. The tourist attractions take place in the wild. | 1. - | |
|  | 1. Evidence of direct conservation benefit | 1. No | 1. Tourism in dolphin habitats may in fact have a negative impact on dolphin conservation. | 1. [115-119] | |
|  | 1. Evidence of indirect benefit | 1. No | 1. - | 1. - | |
| 1. Welfare | 1. Intention to promote / increase welfare? | 1. No | 1. Tourism may have a negative impact on dolphin welfare. | 1. [116] | |

I

|  | 1. **Dolphin sanctuaries** | | | |  |
| --- | --- | --- | --- | --- | --- |
|  | 1. Criterion | 1. Score | 1. Justification / details | 1. Supporting Reference | |
|  | 1. Animals wild or captive? | 1. Captive | 1. - | 1. - | |
| 1. Accessibility | 1. No. animals in attraction | 1. 30 | 1. 24 bottlenose dolphins in the Dolphin Research Centre and 6 bottlenose dolphins in The Dolphin Marine Magic centre. | [120] [121] | |
|  | 1. No. of tourists | 1. 140,000 | 1. The Dolphin research Centre receives ~70,000 visitors per annum and to our knowledge there are two dolphin sanctuaries in the world | 1. [122] | |
| 1. Conservation | 1. IUCN population status | 1. LC | 1. Bottle nose dolphin (*Tursiops truncatus*) (LC) | 1. [108] | |
|  | 1. Animals sourced from the wild? | 1. No | 1. Animals are sourced from captive environments or, if injured beyond the possibility of wild-survival, from the wild. | 1. [120] | |
|  | 1. Evidence of direct conservation benefit | 1. Equivocal | 1. Captive bred dolphins are not reintroduced, but injured wild-caught dolphins may be rehabilitated and released. | 1. [123] | |
|  | 1. Evidence of indirect benefit | 1. Yes | 1. Education and research occur at the dolphin centres. | 1. [124] [125] | |
| 1. Welfare | 1. Intention to promote / increase welfare? | 1. Yes | 1. The intention is to improve the net welfare of the animals through rescue programmes. | 1. [123, 126] | |

J

|  | 1. **Elephant parks** | | | |  |
| --- | --- | --- | --- | --- | --- |
|  | 1. Criterion | 1. Score | 1. Justification / details | 1. Supporting Reference | |
|  | 1. Animals wild or captive? | 1. Captive | 1. - | 1. - | |
| 1. Accessibility | 1. No. animals in attraction | 1. 10,0000-100,000 | 1. Estimated 16,000 captive elephants across Asia and 1,000 African elephants captive worldwide. Many not employed in relevant attractions, but given the widespread nature of these attractions a larger proportion will be. | 1. [127] [128] | |
|  | 1. No. of tourists | 1. >500,000 | 1. 112 relevant elephant attractions are listed on TripAdvisor.com alone. |  | |
| 1. Conservation | 1. IUCN population status | 1. EN-VU | 1. Both Asian (*Elephas maximus*, EN) and African (*Loxodonta africana*, VU) elephants are used in elephant rides | 1. [129, 130] | |
|  | 1. Animals sourced from the wild? | 1. Yes | 1. A large proportion of captive Asian elephants (60%) in Thailand, now principally used in tourism, originated in Myanmar, and at least half of the captive Myanmar population was originally wild caught. Between 2011-13 81 wild elephants were poached for tourism. Therefore at least some poaching of wild elephants, which will ultimately feed into tourism activities, continues. | 1. [131, 132] | |
|  | 1. Evidence of direct conservation benefit | 1. No | 1. - | 1. - | |
|  | 1. Evidence of indirect benefit | 1. No | 1. - | 1. - | |
| 1. Welfare | 1. Intention to promote / increase welfare? | 1. In a minority of cases | 1. A small percentage of elephant parks on TripAdvisor.com take in orphaned or ex-logging elephants, with the intention of providing a better quality of life. The vast majority, however, do not. | 1. - | |

K

|  | 1. **Elephant sanctuaries** | | | |  |
| --- | --- | --- | --- | --- | --- |
|  | 1. Criterion | 1. Score | 1. Justification / details | 1. Supporting Reference | |
|  | 1. Animals wild or captive? | 1. Captive | 1. - | 1. - | |
| 1. Accessibility | 1. No. animals in attraction | 1. 100-200 | 1. 11 qualifying sanctuaries (see Methods) found on TripAdvisor.com have between 3-12 elephants each. | 1. [133, 134] | |
|  | 1. No. of tourists | 1. ~10,000 | 1. Details largely unavailable but one Laotian sanctuary hosts two groups of nine visitors a week (930 per annum). | 1. [29] | |
| 1. Conservation | 1. IUCN population status | 1. VU-EN | 1. Both Asian (*Elephas maximus*, EN) and African (*Loxodonta africana*, VU) elephants. | 1. [129, 130] | |
|  | 1. Animals sourced from the wild? | 1. No | 1. Animals are sourced from other captive attractions. | 1. [133, 134] | |
|  | 1. Evidence of direct conservation benefit | 1. No | 1. Elephants unlikely to be reintroduced after a captive history. | 1. - | |
|  | 1. Evidence of indirect benefit | 1. Yes | 1. Sanctuaries are often part of conservation schemes promoting conservation through education. | 1. [135] | |
| 1. Welfare | 1. Intention to promote / increase welfare? | 1. Yes | 1. The aim of the sanctuaries is to provide better welfare for animals that have been kept in captivity. | 1. [133, 134] | |

L

|  | 1. **Gibbon watching** | | | |  |
| --- | --- | --- | --- | --- | --- |
|  | 1. Criterion | 1. Score | 1. Justification / details | 1. Supporting Reference | |
|  | 1. Animals wild or captive? | 1. Wild | 1. - | 1. - | |
| 1. Accessibility | 1. No. animals in attraction | 1. 8-9 | 1. One group of gibbons is being habituated by the WCS and another family was habituated in 2010 in Veun Sai-Siem. | 1. [136] 2. Wildlife Conservation Society Cambodia, pers. comm. | |
|  | 1. No. of tourists | 1. 1,500 | 1. 6 people per group and day visit the gibbons in Veun Sai-Siem for 8 months of the year. | 1. [32] | |
| 1. Conservation | 1. IUCN population status | 1. EN | 1. Capped gibbon (*Hylobates pileatus*) (EN) | 1. [137] | |
|  | 1. Animals sourced from the wild? | 1. No | 1. Activity is in the wild. | 1. - | |
|  | 1. Evidence of direct conservation benefit | 1. Yes | 1. The local community receive extra funding when visitors spot gibbons, providing an incentive for conservation. Additionally, tourism in the forest provides income for the community promoting its conservation. The Veun Sai-Siem centre works with NGOs towards the conservation of gibbons and their habitat. Increased risk from poaching has not been taken into account, although the project is developing strategies for minimising these risks. | 1. [138] 2. WCS Cambodia, pers. comm. | |
|  | 1. Evidence of indirect benefit | 1. Yes | 1. The activity provides indirect conservation benefits through education of both local communities and visiting tourists. | 1. - | |
| 1. Welfare | 1. Intention to promote / increase welfare? | 1. No | 1. No direct intention to promote welfare | 1. WCS Cambodia, pers. comm. | |

M

|  | 1. **Gorilla watching** | | | |  |
| --- | --- | --- | --- | --- | --- |
|  | 1. Criterion | 1. Score | 1. Justification / details | 1. Supporting Reference | |
|  | 1. Animals wild or captive? | 1. Wild | 1. - | 1. - | |
| 1. Accessibility | 1. No. animals in attraction | 1. Up to 873 | 1. There are approximately 480 gorillas in Rawanda and 393 gorillas in Uganda where gorilla trekking is the most common. | 1. [34, 139] | |
|  | 1. No. of tourists | 1. 58,000 | 1. Approximately 80 visitors per day visit each of the two sites in Rwanda and Uganda. | 1. [34, 139] | |
| 1. Conservation | 1. IUCN population status | 1. EN-CE | 1. *Gorilla beringei* (EN), *Gorilla gorilla* (CE) | 1. [140, 141] | |
|  | 1. Animals sourced from the wild? | 1. No | 1. Animals maintained in the wild. | 1. - | |
|  | 1. Evidence of direct conservation benefit | 1. Yes | 1. Visitor revenues support the conservation of the gorillas and the forests they inhabit. | 1. [139, 142] [34, 143] [144] | |
|  | 1. Evidence of indirect benefit | 1. Yes | 1. Education as part of the tourism in the national reserves provides an indirect conservation benefit. | 1. [139] 2. [142] 3. [34] | |
| 1. Welfare | 1. Intention to promote / increase welfare? | 1. No | 1. Gorillas may be affected by transmitted diseases and injury from interacting with visitors. | 1. [145] [146] [147] | |

N

|  | 1. **Hyena men** | | | |  |
| --- | --- | --- | --- | --- | --- |
|  | 1. Criterion | 1. Score | 1. Justification / details | 1. Supporting Reference | |
|  | 1. Animals wild or captive? | 1. Captive | 1. The hyenas live in captivity with their handlers. | 1. [148] | |
| 1. Accessibility | 1. No. animals in attraction | 1. 3 | 1. The hyena men possess three hyenas. | 1. [35] | |
|  | 1. No. of tourists | 1. >1000 | 1. The hyena men visit towns in Nigeria for a few months every year to sell traditional remedies to the local communities. | 1. [149] | |
| 1. Conservation | 1. IUCN population status | 1. LC | 1. Spotted hyena (*Crocuta crocuta*) (LC) | 1. [150] | |
|  | 1. Animals sourced from the wild? | 1. Yes | 1. The hyenas are caught from the wild. | 1. [148] | |
|  | 1. Evidence of direct conservation benefit | 1. No | 1. - | 1. - | |
|  | 1. Evidence of indirect benefit | 1. No | 1. - | 1. - | |
| 1. Welfare | 1. Intention to promote / increase welfare? | 1. No | 1. The intention is said to be attracting people to buy traditional medicine. | 1. [149] | |

O

|  | 1. **Lion encounters** | | | |  |
| --- | --- | --- | --- | --- | --- |
|  | 1. Criterion | 1. Score | 1. Justification / details | 1. Supporting Reference | |
|  | 1. Animals wild or captive? | 1. Captive | 1. - | 1. - | |
| 1. Accessibility | 1. No. animals in attraction | 1. 1,000-10,000 | 1. 16 lion breeding / handling attractions are listed on TripAdvisor.com. Claims that one attraction alone has > 100 individuals. | 1. [151] | |
|  | 1. No. of tourists | 1. 100,000-500,000 | 1. Tour companies state that they receive a high number of visitors each year. | 1. - | |
| 1. Conservation | 1. IUCN population status | 1. VU | 1. *Pantherea leo* | 1. [152] | |
|  | 1. Animals sourced from the wild? | 1. No | 1. Captive bred | 1. [153] | |
|  | 1. Evidence of direct conservation benefit | 1. No | 1. To date no reintroduction has occurred. Highly questionable that captive breeding of lions is a conservation-relevant activity. | 1. [153] [154] [155] | |
|  | 1. Evidence of indirect benefit | 1. Yes | 1. Attractions do provide education on lion conservation, which may indirectly benefit the species' conservation status. | 1. [151, 154, 155] | |
| 1. Welfare | 1. Intention to promote / increase welfare? | 1. No | 1. - | 1. - | |

P

|  | 1. **Lion sanctuaries** | | | |  |
| --- | --- | --- | --- | --- | --- |
|  | 1. Criterion | 1. Score | 1. Justification / details | 1. Supporting References | |
|  | 1. Animals wild or captive? | 1. Captive | 1. - | 1. - | |
| 1. Accessibility | 1. No. animals in attraction | 1. >213 | 1. The figure is an estimate from the number of lions in six sanctuaries worldwide. | [156-160] | |
|  | 1. No. of tourists | 1. >70,000 | 1. Many sanctuaries have guided tours for children with 20-30 children in each group. | 1. [156, 161] | |
| 1. Conservation | 1. IUCN population status | 1. VU | 1. Lion (*Panthera leo*) (VU) | 1. [152] | |
|  | 1. Animals sourced from the wild? | 1. No | 1. The lions are sourced from captive environments. | 1. - | |
|  | 1. Evidence of direct conservation benefit | 1. No | 1. No re-introduction or breeding take place. | 1. [162] | |
|  | 1. Evidence of indirect benefit | 1. Yes | 1. Education through tours and talks. | 1. [163] | |
| 1. Welfare | 1. Intention to promote / increase welfare? | 1. Yes | 1. The aim of the sanctuaries is to improve the welfare status of captive lions. | 1. [163] | |

Q

|  | 1. **Orang-utan sanctuaries** | | | |  |
| --- | --- | --- | --- | --- | --- |
|  | 1. Criterion | 1. Score | 1. Justification / details | 1. Supporting Reference | |
|  | 1. Animals wild or captive? | 1. Captive | 1. - | 1. - | |
| 1. Accessibility | 1. No. animals in attraction | 1. >100 | 1. 80 orang-utans in one centre in Sepilok, and 23 in Samboja. | [47]Sunderland-Groves, J. pers comm. | |
|  | 1. No. of tourists | 1. 2,000-8,000 | 1. Max. 7 visitors in each group in one sanctuary. If one group every day, this would suggest >2,000 a year. The Samboja sanctuary in Borneo has approximately 500 tourists per year. We have identified four orang-utan sanctuaries. | 1. [164] Sunderland-Groves, J. pers comm. | |
| 1. Conservation | 1. IUCN population status | 1. EN-CR | 1. Sumatran orang-utan (*Pongo abelii*) (CE), Bornean orang-utan (*Pongo pygmaeus*) (E) | 1. [165, 166] | |
|  | 1. Animals sourced from the wild? | 1. No | 1. The animals are sourced from captive environments, or, rarely, rehabilitated from the wild if sick, injured or in danger of persecution. | 1. [167] Sunderland-Groves, J. pers comm. | |
|  | 1. Evidence of direct conservation benefit | 1. Yes | 1. Through restoration schemes replanting lost habitat, and rehabilitation. The Borneo Orangutan Survival Foundation have released 130+ orangutans into the wild since its release camp became operational in 2012. | 1. [168, 169] | |
|  | 1. Evidence of indirect benefit | 1. Yes | 1. Through education. | 1. [170, 171] | |
| 1. Welfare | 1. Intention to promote / increase welfare? | 1. Yes | 1. The orang-utans are sourced from other captive attractions to improve their welfare. | 1. [47] | |

R

|  | **Polar bear watching** | | | |  |
| --- | --- | --- | --- | --- | --- |
|  | 1. Criterion | 1. Score | 1. Justification / details | 1. Supporting Reference | |
|  | 1. Animals wild or captive? | 1. Wild | 1. - | 1. - | |
| 1. Accessibility | 1. No. animals in attraction | 1. 250-500 | 1. Estimate. 1,000 bears are thought to inhabit Churchill, Canada. | 1. [172] | |
|  | 1. No. of tourists | 1. >6,000 | 1. 6,000 per annum in Churchill, Canada. This would increase with inclusion of Greenland and Svalbard. | 1. [173] 2. [174] | |
| 1. Conservation | 1. IUCN population status | 1. VU | 1. Polar bear (*Ursus maritimus*) (VU) | 1. [175] | |
|  | 1. Animals sourced from the wild? | 1. No | 1. The activity is in the wild. | 1. - | |
|  | 1. Evidence of direct conservation benefit | 1. No | 1. There is no evidence that polar bear tourism benefits bear conservation directly. | 1. - | |
|  | 1. Evidence of indirect benefit | 1. Yes | 1. Education may promote indirect conservation of polar bears. | 1. [176] | |
| 1. Welfare | 1. Intention to promote / increase welfare? | 1. No | 1. Polar bears have been approached and harassed by tourists that come too close. | 1. [177] 2. [174] | |

S

|  | 1. **Sea turtle farming** | | | |  |
| --- | --- | --- | --- | --- | --- |
|  | 1. Criterion | 1. Score | 1. Justification / details | 1. Supporting Reference | |
|  | 1. Animals wild or captive? | 1. Captive | 1. - | 1. - | |
| 1. Accessibility | 1. No. animals in attraction | 1. >70,000 | 1. The Cayman turtle farm has 7,000 turtles. La Tortugranja hatchery in Mexico released 67,000 turtles in 2013. | 1. [178, 179] | |
|  | 1. No. of tourists | 1. >500.000 | 1. The Cayman turtle farm receives approximately 500,000 visitors per annum. | 1. [180] | |
| 1. Conservation | 1. IUCN population status | 1. EN | 1. Green turtle (*Chelonia mydas*) | 1. [181] | |
|  | 1. Animals sourced from the wild? | 1. No | 1. The turtles are bred in the farms. | 1. [180] | |
|  | 1. Evidence of direct conservation benefit | 1. Equivocal / no | 1. There is no evidence that the reintroduction of captive bred turtles contributes directly to the conservation of turtles. The effectiveness of supplying farmed turtle meat to reduce consumption of wild-caught turtles is debateable. | 1. [182, 183] | |
|  | 1. Evidence of indirect benefit | 1. Yes | 1. The Cayman turtle farm claims to provide conservation education. | 1. [180] | |
| 1. Welfare | 1. Intention to promote / increase welfare? | 1. No | 1. The turtles hare kept in crowded pools with concomitant risk of injury and disease transmission. | 1. [183] [184] | |

T

|  | 1. **Shark cage diving** | | | |  |
| --- | --- | --- | --- | --- | --- |
|  | 1. Criterion | 1. Score | 1. Justification / details | 1. Supporting Reference | |
|  | 1. Animals wild or captive? | 1. Wild | 1. - | 1. - | |
| 1. Accessibility | 1. No. animals in attraction | 1. ~180 | 1. Shark cage tourism is known to exist in the Bahamas, Fiji, South Africa, Australia and French Polynesia. 36 sharks have been recorded in one location in French Polynesia. If we assume that the tour companies in a country largely use the same area this would be a conservative estimate of the number of animals encountered. | 1. [185] | |
|  | 1. No. of tourists | 1. >1.5 million | 1. The tour companies take approximately 30 visitors per trip, up to three times a day. Eight tour operators have been found in one town in South Africa, Gasbai. As a conservative estimate there may be 10 tour operators in the five locations above with a total of over 1.5 million visitors per annum. | 1. [185] [186] | |
| 1. Conservation | 1. IUCN population status | 1. VU-NT | 1. Great white shark (*Carcharodon carcharias*) (VU); Lemon shark (*Negaprion brevirostris*) (NT) | 1. [187, 188] | |
|  | 1. Animals sourced from the wild? | 1. No | 1. The tourist activity is in the wild. | 1. - | |
|  | 1. Evidence of direct conservation benefit | 1. No | 1. There is no evidence that shark cage tourism has provided direct conservation benefits to shark species or their habitats. | 1. - | |
|  | 1. Evidence of indirect benefit | 1. No | 1. The reverse: chumming (baiting) has been shown to increase inbreeding in sharks due to the aggregation of individuals in the same area. | 1. [185] | |
| 1. Welfare | 1. Intention to promote / increase welfare? | 1. No | 1. Chumming may make sharks dependent on tourism for food which may develop aggression towards humans, leading to incidental disease or injury. | 1. [189] [190] | |

U

|  | 1. **Snake charming** | | | |  |
| --- | --- | --- | --- | --- | --- |
|  | 1. Criterion | 1. Score | 1. Justification / details | 1. Supporting Reference | |
|  | 1. Animals wild or captive? | 1. Captive | 1. - | 1. - | |
| 1. Accessibility | 1. No. animals in attraction | 1. 200-300 | 1. India and Bangladesh are thought to have around 200 snake charmers with at least one snake each. | 1. [191] | |
|  | 1. No. of tourists | 1. 150,000-350,000 | 1. The number of tourists in street performances are difficult to assess, however, if each snake charmer attracts 2-5 tourists per day this would represent 150,000-350,000 visitors annually. | 1. - | |
| 1. Conservation | 1. IUCN population status | 1. LC-VU | 1. King cobra (*Ophiphagus hannah*) (VU); Common bamboo viper (*Trimeresurus gramineus*) (LC); Burmese python (*Python bivittatus*) (VU); Indian rat snake (*Ptyas mucosa*) (not assessed) | 1. [192, 193] [194] | |
|  | 1. Animals sourced from the wild? | 1. Yes | 1. There is little evidence for the source of the snakes. In most cases they are assumed have been directly or indirectly (through a snake dealer) caught from the wild. | 1. [195] | |
|  | 1. Evidence of direct conservation benefit | 1. No | 1. - | 1. - | |
|  | 1. Evidence of indirect benefit | 1. No | 1. - | 1. - | |
| 1. Welfare | 1. Intention to promote / increase welfare? | 1. No | 1. - | 1. - | |

V

|  | **Street dancing macaques** | | | |  |
| --- | --- | --- | --- | --- | --- |
|  | Criterion | Score | Justification / details | Supporting Reference | |
|  | Animals wild or captive? | Captive | - | - | |
| Accessibility | No. animals in attraction | Up to 200 in Jakarta, unknown elsewhere, but likely to be widespread. | Dancing macaques also known to be used in India and Myanmar, and despite ban in Jakarta, the practice is likely to occur in Indonesia and south east Asia. | [196, 197]  N. D'Cruze, pers. obs. | |
|  | No. of tourists | Unknown, but likely to be substantial. | Although practice now banned in Jakarta (since 2013), macaques are not protected in Indonesia, so it may still occur in other cities in Indonesia and south east Asia. Macaques are used to attract passers-by, so a large audience potentially still remains. | [196]N. D'Cruze, pers. obs. | |
| Conservation | IUCN population status | LC | Attraction principally uses long tailed macaques (*Macaca fascicularis*), but rhesus macaque also used (*Macaca mulatta*) | [196]  N. D'Cruze, pers. obs. | |
|  | Animals sourced from the wild? | Yes | - | [196, 197] | |
|  | Evidence of direct conservation benefit | No | - | - | |
|  | Evidence of indirect benefit | No | No educational benefit. | - | |
| Welfare | Intention to promote welfare? | No | - | - | |

W

|  | 1. **Tiger farms** | | | |  |
| --- | --- | --- | --- | --- | --- |
|  | 1. Criterion | 1. Score | 1. Justification / details | 1. Supporting Reference | |
|  | 1. Animals wild or captive? | 1. Captive | 1. - | 1. - | |
| 1. Accessibility | 1. No. animals in attraction | 1. 5,000 | 1. There are approximately 200 tiger farms in China housing ca 5,000 tigers, as well as farms in Lao PDR and Thailand. | 1. [69] N. D'Cruze pers. obs. | |
|  | 1. No. of tourists | 1. >50,000 | 1. Hundreds of tourists per day are thought to visit one tiger farm in China alone. | 1. [198] | |
| 1. Conservation | 1. IUCN population status | 1. EN | 1. Tiger (*panthera tigris*) (EN) | 1. [199] | |
|  | 1. Animals sourced from the wild? | 1. No | 1. Tigers are bred in captivity. | 1. [200] | |
|  | 1. Evidence of direct conservation benefit | 1. No | 1. Tiger farms are sometimes claimed to reduce pressure on wild populations, but high demand remains for wild-caught tiger products. | 1. [200, 201] | |
|  | 1. Evidence of indirect benefit | 1. No | 1. - | 1. - | |
| 1. Welfare | 1. Intention to promote / increase welfare? | 1. No | 1. - | 1. - | |

X

|  | Tiger interactions | | | |  |
| --- | --- | --- | --- | --- | --- |
|  | Criterion | Score | Justification / details | Supporting Reference | |
|  | Animals wild or captive? | Captive | - | - | |
| Accessibility | No. animals in attraction | 200-600 | 621 tigers recorded in Thailand, housed in 10 venues, 8 of which provide photo opportunities and 5 cub feeding. 135 known specifically at the Kanchanaburi Tiger Temple, At least 60 at Tiger Kingdom Ubon, unknown at Tiger Kingdom Phuket and Chiang Mai. | [202, 203] | |
|  | No. of tourists | >300,000 p/a | Tiger Temple received between 300-880 tourists per day in 2007-2008. Numbers for Tiger Kingdom attractions unknown but likely to be similar. | [204] | |
| Conservation | IUCN population status | EN | - | [199] | |
|  | Animals sourced from the wild? | No | Tigers bred in zoos, but also in farms outside of Thailand. | [204] | |
|  | Evidence of direct conservation benefit | No | No evidence of successful (or attempted) reintroduction of tigers from any of these attractions. | - | |
|  | Evidence of indirect benefit | No | Attractions do not promote education of tourists in welfare / conservation. | - | |
| Welfare | Intention to promote / increase welfare? | No | - | - | |
