# Supplementary material for: The Customer Isn't Always Right—Conservation and Animal Welfare Implications of the Increasing Demand for Wildlife Tourism
Source: PLoS One. 2015 Oct 21;10(10):e0138939. doi: 10.1371/journal.pone.0138939 (PMC4619427; doi:10.1371/journal.pone.0138939)
Supplement: S4 Table — Scores are based on the degree to which the five freedoms of captive animals are fulfilled. A score of 1 indicates that a give freedom was likely to be fulfilled by all WTAs within a given WTA type; conversely a score of 0 indicates that all WTAs were unlikely to fulfil the freedom. A score of <1 indicates that WTAs within a given type may vary in the extent to which they meet this freedom. WTA types are listed alphabetically. See S2 Appendix for reference citations. (DOCX) [file pone.0138939.s007.docx]

**S4 Table**

A

| **Bear bile farms** | | | |  |
| --- | --- | --- | --- | --- |
| Freedom | Score | Justification | Supporting References | |
| From hunger/thirst | 0 | Typically emaciated with no ad-lib access to water. | [205] | |
| From discomfort | 0 | Permanently catheterised; cages too small. | [205] | |
| From pain/injury/disease | 0 | Teeth worn / broken; constant rubbing; frequent self mutilation. | [205] | |
| To behave normally | 0 | Confined in small cages; stereotypical behaviours common; permanent behavioural abnormalities. | [205] | |
| From fear and distress | 0 | Expression of aggression / nervousness / agitation. | [205] | |
| Total | 0 |  |  | |

B

| 1. **Bear dancing** | | | |  |
| --- | --- | --- | --- | --- |
| 1. Freedom | 1. Score | 1. Justification / details | 1. Supporting References | |
| 1. From hunger/thirst | 1. <1 | 1. Bears fed a diet rich in human (potentially unsuitable) foods (bread, rice, ghee) | 1. [82] | |
| 1. From discomfort | 1. 0 | 1. Bears maintained attached to bamboo pole via rope through nose or cheek. | 1. [82] | |
| 1. From pain/injury/disease | 1. 0 | 1. See above. Also canine teeth and claws often removed. | 1. [82] | |
| 1. To behave normally | 1. 0 | 1. See above. Training for dancing requires aversion training, involving burning the bears’ paws. | 1. [82] [10] | |
| 1. From fear and distress | 1. 0 | 1. See above | 1. [82] | |
| 1. Total | 1. <1 |  |  | |

C

| **Bear parks** | | | |  |
| --- | --- | --- | --- | --- |
| Freedom | Score | Justification / details | Supporting References | |
| From hunger/thirst | 0 | Bears not fed during day, to encourage begging. Diet ad-hoc, provided by donations, and often unsuitable. | [87, 206] | |
| From discomfort | 0 | Bears maintained in concrete pits in overcrowded conditions. | [85] | |
| From pain/injury/disease | <1 | Bears often injured. | [87] | |
| To behave normally | 0 | Boredom during confinement (maintained in concrete pits). Bears must beg for food. Bear entertainment shows include basketball, soccer and mathematics on stage. | [11, 85] | |
| From fear and distress | 0 | Overcrowding, boredom and stress can lead to fights. | [87] | |
| Total | <1 |  |  | |

D

| 1. **Bear sanctuaries** | | | |  |
| --- | --- | --- | --- | --- |
| 1. Freedom | 1. Score | 1. Justification / details | 1. Supporting Reference | |
| 1. From hunger/thirst | 1. 1 | 1. Standards may vary between sanctuaries, but we assume that nutritional requirements are adequately met. | 1. [12] | |
| 1. From discomfort | 1. 1 | 1. We assume that husbandry standards are adequately met, especially in sanctuaries providing large forested areas. | 1. [12] | |
| 1. From pain/injury/disease | 1. 1 | 1. We assume that veterinary and husbandry standards are adequately met. | 1. [12] | |
| 1. To behave normally | 1. <1 | 1. Although some sanctuaries provide large forested areas, other captive environments may reduce the ability of animals to behave normally. | 1. [12] | |
| 1. From fear and distress | 1. 1 | 1. The fulfilment of the above suggests that the animals are relatively free from distress and fear. | 1. - | |
| 1. Total | 1. <5 |  |  | |

E

| 1. **Civet coffee farms** | | | |  |
| --- | --- | --- | --- | --- |
| 1. Freedom | 1. Score | 1. Justification / details | 1. Supporting Reference | |
| 1. From hunger/thirst | 1. 0 | 1. Disproportionate provisioning of coffee cherries in diet, other needs not sufficiently met. | 1. [91, 207, 208] | |
| 1. From discomfort | 1. 0 | 1. Fur loss and injuries common. | 1. [91, 207, 208] | |
| 1. From pain/injury/disease | 1. 0 | 1. Confinement stress and insufficient meeting of dietary requirements can lead to disease and injury. | 1. [91, 208] | |
| 1. To behave normally | 1. 0 | 1. Stereotypical behaviours evident. Inappropriate housing. | 1. [208] | |
| 1. From fear and distress | 1. 0 | 1. Confinement with / close to other civets can cause fights and distress. | 1. [91] | |
| 1. Total | 1. 0 |  |  | |

F

| 1. **Crocodile farms** | | | |  |
| --- | --- | --- | --- | --- |
| 1. Freedom | 1. Score | 1. Justification / details | 1. Supporting Reference | |
| 1. From hunger/thirst | 1. <1 | 1. Farms in some developing countries may not be able to provide suitable food because meat is expensive. | 1. [93] | |
| 1. From discomfort | 1. 0 | 1. The close proximity to other crocodiles may make it difficult for them to rest in the same way as in the wild. | 1. [209] | |
| 1. From pain/injury/disease | 1. <1 | 1. Although it is in the keepers’ interest to have healthy animals, salmonella and other diseases have been found in the crocodile meat. | 1. [100] | |
| 1. To behave normally | 1. 0 | 1. The crowded environment is unlikely to allow the crocodiles to behave normally. | 1. - | |
| 1. From fear and distress | 1. <1 | 1. If many crocodiles are placed together the loss of territorial space may result in aggression and reduced breeding success. | 1. [209] | |
| 1. Total | 1. <3 |  |  | |

G

| 1. **Dolphin interactions (captive)** | | | |  |
| --- | --- | --- | --- | --- |
| 1. Freedom | 1. Score | 1. Justification / details | 1. Supporting References | |
| 1. From hunger/thirst | 1. 1 | 1. We assume that nutritional requirements are adequately met. | 1. - | |
| 1. From discomfort | 1. 0 | 1. Captive environment and repeated human interaction may result in ill effects from direct injury, sunburn, paint, chlorine. | 1. [105, 210, 211] | |
| 1. From pain/injury/disease | 1. <1 | 1. Captive stress and repeated contact with humans often lead to injury and disease, but veterinary assistance may at least partially compensate. | 1. [210] [105] | |
| 1. To behave normally | 1. 0 | 1. Confined in pools inadequate to meet basic social needs. | 1. [210] [105] | |
| 1. From fear and distress | 1. <1 | 1. Unsuitable or unstable social environment can lead to aggression, resulting in distress (sometimes resulting in mortality). | 1. [210] [105] | |
| 1. Total | 1. <3 |  |  | |

H

| 1. **Dolphin interactions (wild)** | | | |  |
| --- | --- | --- | --- | --- |
| 1. Freedom | 1. Score | 1. Justification / details | 1. Supporting Reference | |
| 1. From hunger/thirst | 1. 1 | 1. Tourism probably does not impact on the dolphins' ability to feed. | 1. - | |
| 1. From discomfort | 1. 1 | 1. Although dolphins may not be able to completely escape tourism they are able to avoid swimming visitors. | 1. [113] | |
| 1. From pain/injury/disease | 1. <1 | 1. The close interaction with visitors may cause injury and disease. | 1. [211] | |
| 1. To behave normally | 1. <1 | 1. The presence of tourists may impact on the ability for dolphins to create social bonds and reproduce. | 1. [212] | |
| 1. From fear and distress | 1. <1 | 1. The presence of tourists may cause distress, shown by avoidance behaviour in response to the presence of divers. | 1. [116] [113] | |
| 1. Total | 1. < 5 |  |  | |

I

| 1. **Dolphin sanctuaries** | | | |  |
| --- | --- | --- | --- | --- |
| 1. Freedom | 1. Score | 1. Justification / details | 1. Supporting Reference | |
| 1. From hunger/thirst | 1. 1 | 1. The dolphins are said to be fed good quality fish. | [126] | |
| 1. From discomfort | 1. 1 | 1. The dolphins are kept in salt water pools that provide a better habitat than chlorinated pools. | 1. [213] | |
| 1. From pain/injury/disease | 1. 1 | 1. We assume that veterinary standards and handling are appropriate. | 1. [126, 214] | |
| 1. To behave normally | 1. 0 | 1. Dolphins are held captive in pools and so unable to occupy the ranges that they would in the wild. Additionally they have limited exposure to other dolphins and may not interact socially as they would in the wild. | 1. [120] | |
| 1. From fear and distress | 1. <1 | 1. The dolphins in some sanctuaries may be used in shows and dolphin interactions, which can cause distress in captive dolphins in some settings. | 1. [215, 216] | |
| 1. Total | 1. < 4 |  |  | |

J

| 1. **Elephant parks** | | | |  |
| --- | --- | --- | --- | --- |
| 1. Freedom | 1. Score | 1. Justification / details | 1. Supporting References | |
| 1. From hunger/thirst | 1. <1 | 1. Many attractions struggle to provide sufficient variety in diet. | 1. [217] | |
| 1. From discomfort | 1. <1 | 1. Basic shelter needs often met, but protracted periods chained. | 1. [218] | |
| 1. From pain/injury/disease | 1. 0 | 1. Frequently brutal initial training designed to instil a fear of pain. Injuries from use of bullhook common. | 1. [128, 217, 218] | |
| 1. To behave normally | 1. 0 | 1. Artificial social systems may give rise to behavioural stress. Repeated and obligatory interaction with tourists. Protracted periods chained. | 1. [217] | |
| 1. From fear and distress | 1. 0 | 1. Harsh training methods instil fear of handlers. | 1. [128, 217, 218] | |
| 1. Total | 1. <2 |  |  | |

K

| **Elephant sanctuaries** | | | |  |
| --- | --- | --- | --- | --- |
| 1. Freedom | 1. Score | 1. Justification / details | 1. Supporting References | |
| 1. From hunger/thirst | 1. 1 | 1. We assume that nutritional requirements will be adequately met. | 1. [219] | |
| 1. From discomfort | 1. 1 | 1. Sanctuaries specifically aim to provide a comfortable environment, and do not, for example, permit elephant rides and public performances. | 1. [219] | |
| 1. From pain/injury/disease | 1. 1 | 1. We assume that adequate veterinary assistance is provided and animals are not injured by handlers (e.g. through use of bull-hook), although this information is not readily available. | 1. - | |
| 1. To behave normally | 1. <1 | 1. Although we expect that there will be a net improvement in the ability for elephants to behave normally, wild home ranges may be up to 37 km^2^ , which is unlikely to be achieved in (semi) captivity. | 1. [220] [219] | |
| 1. From fear and distress | 1. 1 | 1. The above scores suggest that elephant sanctuaries provide environments relatively free from fear and distress. | 1. - | |
| 1. Total | 1. <5 |  |  | |

| LGibbon watching | | | |  |
| --- | --- | --- | --- | --- |
| Freedom | Score | Justification / details | Supporting References | |
| From hunger/thirst | 1 | The tourism does not impact on feeding as the gibbons are in the canopy away from the visitors. | - | |
| From discomfort | 1 | As the gibbons stay in the canopy the risk of this is low. | [136] | |
| From pain/injury/disease | 1 | The visitors do not come into contact with the gibbons and so there no additional risk from the project of injury or disease transmission. | [136] | |
| To behave normally | 1 | The conservation project allows the gibbons to behave naturally. They spend the majority of their time in the canopy. | World Conservation Society Cambodia, pers. comm. | |
| From fear and distress | 1 | Gibbons will be habituated over a year before visitors are introduced, theoretically minimising fear and distress. | World Conservation Society Cambodia, pers. comm. | |
| Total | 5 |  |  | |

M

| Gorilla watching | |  |  |  |
| --- | --- | --- | --- | --- |
| Freedom | Score | Justification / details | Supporting References | |
| From hunger/thirst | 1 | Tourism is not thought to impact on the ability for the gorillas to feed as the visitors stay for a maximum of one hour. | [221] | |
| From discomfort | 1 | Mountain gorillas used for tourism are habituated and may choose to move away from the visitors, who are encouraged to respect the population. | [139] | |
| From pain/injury/disease | <1 | Close human interaction with gorillas may cause injury or transmit diseases from humans to gorillas. Although, visitors must stay 7 m from the gorillas many cases of direct contact have been recorded. | [147][139, 145] | |
| To behave normally | 1 | The short period that the tourists spend with the gorillas (one hour) is not thought to have an impact on their ability to behave normally. | [139, 142] [34] | |
| From fear and distress | 1 | As the gorillas are habituated this suggests that they do not experience fear or distress in the presence of humans. | - | |
| Total | <5 |  |  | |

N

**Hyena men**

| Freedom | Score | Justification / details | Supporting References |
| --- | --- | --- | --- |
| From hunger/thirst | 1 | The hyenas are cared for in the homes of the hyena men as they must be fit to perform in order to bring in people to sell their remedies. | [149] |
| From discomfort | <1 | The hyenas are chained at all times, but have their own sheltered area where they are able to rest. | [149] |
| From pain/injury/disease | 1 | As the men directly depend on the hyenas they are likely to take sufficient care of them. | [149] |
| To behave normally | 0 | Hyenas typically chained, and so unable to behave normally. | - |
| From fear and distress | <1 | Hyena welfare appears generally good, and so they may not experience fear or distress - although this may occur at the point of initial capture and training. | - |
| Total | <4 |  |  |

O

| 1. **Lion encounters** | | | |  |
| --- | --- | --- | --- | --- |
| 1. Freedom | 1. Score | 1. Justification / details | 1. Supporting References | |
| 1. From hunger/thirst | 1. 1 | 1. We assume that nutritional requirements are adequately met. | 1. - | |
| 1. From discomfort | 1. <1 | 1. Repeated disturbance of cubs and sub-adults for tourist activities. | 1. [222] | |
| 1. From pain/injury/disease | 1. <1 | 1. Lions may be physically coerced during walks / handling. | 1. [222, 223] | |
| 1. To behave normally | 1. 0 | 1. Lions human habituated, cubs taken from mother at young age for hand rearing. Individuals typically accompanied by handlers and tourists for much of day. | 1. [223] | |
| 1. From fear and distress | 1. <1 | 1. Early separation from mother may result in distress. | 1. [222] | |
| 1. Total | 1. <4 |  |  | |

P

| 1. **Lion sanctuaries** | | | |  |
| --- | --- | --- | --- | --- |
| 1. Freedom | 1. Score | 1. Justification / details | 1. Supporting References | |
| 1. From hunger/thirst | 1. 1 | 1. Appropriate quantities of meat-based meals are provided. | [224] | |
| 1. From discomfort | 1. 1 | 1. The sanctuaries aim to ensure that the conditions are as good as possible. | 1. [225] | |
| 1. From pain/injury/disease | 1. 1 | 1. In some sanctuaries a team is dedicated to monitoring the well-being of the animals. | 1. [226] | |
| 1. To behave normally | 1. <1 | 1. As the animals are captive they will be unable to behave fully normally. | 1. - | |
| 1. From fear and distress | 1. 1 | 1. We assume that the care provided will not induce fear and distress in the captive lions. | 1. - | |
| 1. Total | 1. <5 |  |  | |

Q

| 1. **Orang-utan sanctuaries** | | | |  |
| --- | --- | --- | --- | --- |
| 1. Freedom | 1. Score | 1. Justification / details | 1. Supporting References | |
| 1. From hunger/thirst | 1. 1 | 1. The orang-utans are fed a basic diet to encourage them to forage themselves, hence providing a natural diet. Samboja islands orang-utans feed within natural vegetation but diet is subsidised by drop feeding. | [47, 227] | |
| 1. From discomfort | 1. <1 | 1. The orang-utans are kept in large enclosures, or free roaming in natural vegetation within defined limits, in most sanctuaries enabling them to keep away from visitors if they choose. In Sumatra visitors must maintain a distance of 10 m when the animals are present. In one sanctuary in Matang Sarawak, Malaysia, however, the orang-utans are kept in small cages. | 1. [47] [164] 2. [169, 227, 228] | |
| 1. From pain/injury/disease | 1. 1 | 1. Medical care is available for the orang-utans and visitors are strictly forbidden to touch or approach them. | 1. [47, 164] | |
| 1. To behave normally | 1. <1 | 1. Most animals live in a large rainforest enclosure, or in vegetated islands, and so have the ability to forage and interact with each other without the presence of visitors. Those in cages, however, may find this freedom limited. | 1. [47, 227, 228] | |
| 1. From fear and distress | 1. 1 | 1. The fulfilment of the above suggests that the orang-utans are free from fear and distress. | 1. - | |
| 1. Total | 1. < 5 |  |  | |

R

| 1. **Polar bear watching** | | | |  |
| --- | --- | --- | --- | --- |
| 1. Freedom | 1. Score | 1. Justification / details | 1. Supporting References | |
| 1. From hunger/thirst | 1. 1 | 1. The polar bears feed very little during the period that the tourism takes place (Oct-Nov). | 1. [229] | |
| 1. From discomfort | 1. <1 | 1. Disturbance by vehicles, 15 per day, at a time when polar bears are dependent on rest. | 1. [229] 2. [174] | |
| 1. From pain/injury/disease | 1. <1 | 1. Vehicles are kept an average of 20 m from the bears, but the presence of humans may increase the risk of disease transmission, and the bears may be killed if demonstrating aggression towards tourists. | 1. [173] 2. [174] | |
| 1. To behave normally | 1. <1 | 1. There is no evidence that suggests that the bears are not able to behave normally, but see below. | 1. [229] 2. [174] | |
| 1. From fear and distress | 1. 0 | 1. Vehicle disturbance may increase vigilance behaviour and metabolic rates of polar bears. | 1. [229] 2. [174] | |
| 1. Total | 1. <4 |  |  | |

S

| **Sea turtle farming** | | | |  |
| --- | --- | --- | --- | --- |
| Freedom | Score | Justification / details | Supporting References | |
| From hunger/thirst | 1 | We assume that nutritional requirements are adequately met, especially as many turtles are bred for meat. | [178] | |
| From discomfort | 0 | Crowded conditions and regular handling by tourists suggest that this freedom is not met. | [183] | |
| From pain/injury/disease | 0 | Injury due to overcrowding and the presence of salmonella and other diseases have been recorded in the pool water. | [184] | |
| To behave normally | 0 | Overcrowded pools and regular handling suggest that the turtles would not be able to behave normally. | - | |
| From fear and distress | 0 | All the above factors may suggest that the turtles are not free from distress. | - | |
| Total | 1 |  |  | |

T

| 1. **Shark cage diving** | | | |  |
| --- | --- | --- | --- | --- |
| 1. Freedom | 1. Score | 1. Justification / details | 1. Supporting References | |
| 1. From hunger/thirst | 1. <1 | 1. Baiting may reduce the time sharks spend hunting as they are attracted to bait (sich oil and blood) thrown overboard to attract them. | 1. [190] | |
| 1. From discomfort | 1. 1 | 1. The activity does not appear to impact on the comfort of the sharks. | 1. - | |
| 1. From pain/injury/disease | 1. 0 | 1. If sharks associate humans with food they may become more aggressive – one result is that sharks may come close to the cages and/or try to break through the metal, increasing the likelihood of injury or disease. | 1. [190] | |
| 1. To behave normally | 1. <1 | 1. As the activity is not chasing the sharks but attracting them to the boat the sharks are allowed to behave normally. However, studies have shown that the feeding behaviour may change due to this activity. | 1. [185] | |
| 1. From fear and distress | 1. 1 | 1. There is no evidence that the activity causes fear or distress (except, potentially, for tourists). | 1. - | |
| 1. Total | 1. <4 |  |  | |

U

| 1. **Snake charming** | | | |  |
| --- | --- | --- | --- | --- |
| 1. Freedom | 1. Score | 1. Justification / details | 1. Supporting References | |
| 1. From hunger/thirst | 1. <1 | 1. The quality or frequency of their feed is unknown but some snakes have their mouths sewn up to protect the handler from being bitten. | 1. [230] 2. [231] | |
| 1. From discomfort | 1. 0 | 1. Snake charmers often travel significant distance to entertain crowds. Protracted confinement for travelling may create uncomfortable conditions. | 1. [191] | |
| 1. From pain/injury/disease | 1. 0 | 1. Snake charmers are thought to treat snakes as disposable, typically consecutively owning seven snakes in a year, suggesting these needs will not be met. | 1. [195] | |
| 1. To behave normally | 1. 0 | 1. The captive conditions suggest that the snakes will be unable to behave normally. | 1. - | |
| 1. From fear and distress | 1. 0 | 1. The attitude that the snakes assume when raising their heads (in response to vibrations) may suggest that they are protecting themselves against aggression. | 1. [231] | |
| 1. Total | 1. <1 |  |  | |

V

| **Street dancing macaques** | | | | |
| --- | --- | --- | --- | --- |
| Freedom | | Score | Justification / details | Supporting References |
| From hunger/thirst | | 1 | Diet assumed adequate, despite uncertainty about whether provisioning is sufficient during performance – no photographs of performance show food or water. | - |
| From discomfort | | <1 | Chained and forced to wear mask. | [196, 197] |
| From pain/injury/disease | | 0 | Chains often grow into skin causing infections and tetanus. Canine teeth pulled. | [196, 197] |
| To behave normally | | 0 | Maintained in cages, perform on streets, kept separate from conspecifics (social animals) | [196, 197] |
| From fear and distress | | 0 | Likely to be beaten during performance / training. Canine teeth pulled. Kept separate from conspecifics (social animals). | [196, 197] |
| Total | | <2 |  |  |

W

| Tiger farm | | | |
| --- | --- | --- | --- |
| Freedom | Score | Justification / details | Supporting Reference |
| From hunger/thirst | 0 | Tigers observed to be malnourished. | [69, 198] |
| From discomfort | 0 | The tigers are maintained permanently in cages that are insufficient to meet their needs. | [232] |
| From pain/injury/disease | 0 | Tigers are unlikely to be free of pain, injury and disease. | [232] |
| To behave normally | 0 | The tigers are unlikely to be able to behave normally. | - |
| From fear and distress | 0 | Due to the above, the participation by captive tigers in shows/performances for tourists and their fate as a product, they are likely to experience fear and distress. | [232] |
| Total | 0 |  |  |

X

| 1. **Tiger interactions** | | | |
| --- | --- | --- | --- |
| 1. Freedom | 1. Score | 1. Justification / details | 1. Supporting References |
| 1. From hunger/thirst | 1. <1 | 1. Diet is often unsuitable. Water inadequately supplied at the majority of venues. | 1. [44, 204] |
| 1. From discomfort | 1. 0 | 1. Cages “barren”. Tiger Temple tigers chained closely to concrete bowls when interacting with public. Tiger Kingdom tigers remain in cages for interaction. Insufficient shade. Sprayed with tiger urine as a compliance measure. Constant interaction with tourists and physical coercion. | 1. [44, 204] |
| 1. From pain/injury/disease | 1. 0 | 1. Tigers trained and handled using physically coercive methods (beaten with sticks, punched, dragged by the tail). Reports from the public of physical abuse and injuries to tigers. | 1. [204] |
| 1. To behave normally | 1. 0 | 1. Constant, obligatory interaction with tourists. Stereotypical behaviours commonplace. Cages insufficient size. No enrichment provided. | 1. [44, 204] |
| 1. From fear and distress | 1. 0 | 1. Above factors may often result in fear or distress. | 1. [204] |
| 1. Total | 1. <1 |  |  |
